# Pollinators drive floral evolution in an Atlantic Forest genus

**DOI:** 10.1093/aobpla/plaa046

**Published:** 2020-08-22

**Authors:** Beatriz Neves, Igor M Kessous, Ricardo L Moura, Dayvid R Couto, Camila M Zanella, Alexandre Antonelli, Christine D Bacon, Fabiano Salgueiro, Andrea F Costa

**Affiliations:** 1 Universidade Federal do Rio de Janeiro, Museu Nacional, Programa de Pós Graduação em Ciências Biológicas (Botânica), São Cristóvão, Rio de Janeiro, RJ, Brazil; 2 Gothenburg Global Biodiversity Centre, SE Gothenburg, Sweden; 3 National Institute of Agricultural Botany, Cambridge, UK; 4 Royal Botanic Gardens, Surrey, UK; 5 Department of Biological and Environmental Sciences, University of Gothenburg, SE Gothenburg, Sweden; 6 Departamento de Botânica, Universidade Federal do Estado do Rio de Janeiro, Rio de Janeiro, RJ, Brazil; 7 Departamento de Botânica, Universidade Federal do Rio de Janeiro, Museu Nacional, São Cristóvão, Rio de Janeiro, RJ, Brazil

**Keywords:** Atlantic Forest, chiropterophily, floral evolution, ornithophily, pollination syndromes, speciation, *Vriesea*

## Abstract

Pollinators are important drivers of angiosperm diversification at both micro- and macroevolutionary scales. Both hummingbirds and bats pollinate the species-rich and morphologically diverse genus *Vriesea* across its distribution in the Brazilian Atlantic Forest. Here, we (i) determine if floral traits predict functional groups of pollinators as documented, confirming the pollination syndromes in *Vriesea* and (ii) test if genetic structure in *Vriesea* is driven by geography (latitudinal and altitudinal heterogeneity) or ecology (pollination syndromes). We analysed 11 floral traits of 58 *Vriesea* species and performed a literature survey of *Vriesea* pollination biology. The genealogy of haplotypes was inferred and phylogenetic analyses were performed using chloroplast (*rps16-trnk* and *matK*) and nuclear (*PHYC*) molecular markers. Floral traits accurately predict functional groups of pollinators in *Vriesea*. Genetic groupings match the different pollination syndromes. Species with intermediate position were found between the groups, which share haplotypes and differ morphologically from the typical hummingbird- and bat-pollinated flowers of *Vriesea*. The phylogeny revealed moderately to well-supported clades which may be interpreted as species complexes. Our results suggest a role of pollinators driving ecological isolation in *Vriesea* clades. Incipient speciation and incomplete lineage sorting may explain the overall low genetic divergence within and among morphologically defined species, precluding the identification of clear species boundaries. The intermediate species with mixed floral types likely represent a window into shifts between pollinator syndromes. This study reports the morphological-genetic continuum that may be typical of ongoing pollinator-driven speciation in biodiversity hotspots.

## Teaser

In this study, we show floral traits to accurately predict hummingbird and bat pollinators in the Atlantic Forest genus *Vriesea*. Genetic groupings match the different pollination syndromes. We identify species with intermediate genetic position which differ morphologically from the typical hummingbird- and bat-pollinated *Vriesea* flowers by their mixed floral traits. We suggest a role of pollinators driving ecological isolation in *Vriesea* clades and that intermediate species likely represent a window into shifts between pollinators.

## Introduction

Ever since the Origin of Species (Darwin 1859), biologists have debated the processes responsible for the generation of morphological and genetic diversity within and among species. Both geographic and ecological factors can shape genetic structure by affecting gene flow through isolation by distance or isolation by environment, respectively. Isolation by distance results from physical, geographic distance and barriers that restrict gene flow among populations (Wright 1943), whereas isolation by environment results from different ecological environments limiting gene flow (Wang and Bradburd 2014). Isolation by environment can be generated by diverse ecological processes, such as pollinator-driven genetic differentiation in plant populations (Johnson 2010). Pollinators are important drivers of angiosperm diversification at both micro- and macroevolutionary scales, either promoting selection of floral traits or speciation (Van der Niet *et al.* 2014).

Bromeliads are a highly diverse angiosperm clade in the Neotropics (Givnish 2017). Specifically, the genus *Vriesea* is one of the most conspicuous representatives of epiphytes in the Atlantic Forest, which constitutes its main centre of diversity (ca. 227 species; Stehmann *et al.* 2009; Ramos *et al.* 2019; Gouda *et al.* 2020, continuously updated), 85 % of which are found in no other biome (BFG - The Brazil Flora Group 2018). Key innovations such as leaves that form a rosette, absorptive trichomes, epiphytism and vertebrate pollination are likely linked to its rapid diversification in the last 6 million years (Givnish *et al.* 2014; Kessous *et al.* 2020). In addition, the genus is distributed across a broad range of habitats in the Atlantic Forest domain, from the restingas (sandy, coastal plains) to campos de altitude (high altitude fields, including cloud forests) and rocky outcrops (inselbergs) (Smith and Downs 1977; Costa *et al.* 2014, 2015; Givnish *et al.* 2014; Ramos *et al.* 2019; Kessous *et al.* 2020). The large latitudinal (ca. 5–30°S) and altitudinal (0–2892 m a.s.l.) amplitude of the Atlantic Forest also make it a prime study area for plant diversification (Antonelli and Sanmartín 2011; Alvares *et al.* 2013). Such extraordinary environmental heterogeneity has been shown to influence *Vriesea* species diversification and variation in morphological traits (Neves *et al.* 2019).

Based on flower morphology, two sections were recognized in *Vriesea*: *V.* sect. *Vriesea*, with red to yellow floral bracts, tubular flowers with exserted stamens; and *V.* sect. *Xiphion* (see Barfuss *et al.* 2016 for the correct application of the name *Xiphion*), with brown to green floral bracts, campanulate corollas with included stamens (Costa *et al.* 2014). These sections were originally described based on two main floral types: hummingbird- and bat-pollinated flowers, respectively (Mez 1896, 1934–1935). Since then, species have been classified into one or another section based on the labile character of the stamen position in relation to the corolla fauce (included vs. exserted) (Mez 1896, 1934–1935; Harms 1930; Smith and Downs 1977). This classification led to the controversial taxonomic placement of some *Vriesea* species with mixed floral traits. To date, no phylogeny supports the two sections as monophyletic (Costa *et al.* 2015; Gomes-da-Silva and Souza-Chies 2017; Machado *et al.* 2020).

The unique set of floral traits (morphology, colour, scent, rewards and phenology) that are associated to a particular group of pollinators define plant pollination syndromes (Fenster *et al.* 2004). Studies on pollination biology of *Vriesea* species from both sections have added information on flower shape, time of anthesis (diurnal or nocturnal), floral visitors and effective pollinators confirming the evident adaptation to hummingbird or bat pollinators (e.g. Sazima *et al.* 1999; Buzato *et al.* 2000). Based on the colours of the corolla and floral bracts and time of flower anthesis, more *Vriesea* species have the hummingbird (ca. 137 spp.) than bat pollination syndrome (ca. 90 spp.) (Costa *et al.* 2014; Gouda *et al.* 2020, continuously updated). This difference in species richness is also seen in the high diversity of Atlantic Forest plant-pollinating hummingbird species compared with that of bats (Grantsau 1989; Marinho-Filho and Sazima 1998). More importantly, bromeliads are the most representative family of hummingbird- and bat-pollinated flora in the Atlantic Forest, with genus *Vriesea* being the richest bat-pollinated lineage (Sazima *et al.* 1999; Buzato *et al.* 2000; Aguilar-Rodriguez *et al.* 2019).

Here, we test if floral traits accurately predict the different functional groups of pollinators in *Vriesea* by using data on known pollinators from the literature. We also test if genetic structure in *Vriesea* is mainly shaped by geography (latitudinal and altitudinal heterogeneity) or ecology (pollination syndromes). Our study provides a first general assessment of the role of pollinator interactions in shaping genetic structure and promoting floral traits diversity in *Vriesea*.

## Materials and Methods

### Morphological analyses of floral traits—predicting functional groups of pollinators and validating pollination syndromes

**Figure 1.**
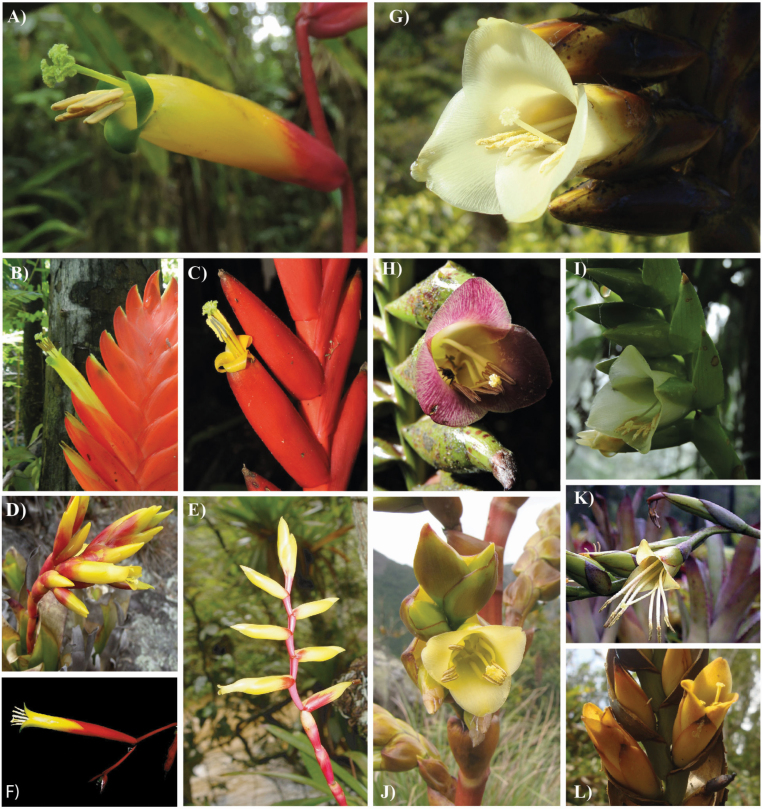
Floral morphological diversity of *Vriesea* species evidencing hummingbird (A–F) and bat (G–L) pollination syndromes. (A) *Vriesea simplex*, (B) *V. duvaliana*, (C) *V. ensiformis* var. *bicolor*, (D) *V. cacuminis*, (E) *V. gracilior*, (F) *V. procera*, (G) *V. pseudatra*, (H) *V. sazimae*, (I) *V. unilateralis*, (J) *V. crassa*, (K) *V. sincorana*, (L) *V. longiscapa*. Photos: B. Neves, F. P. Uribbe, R. L. Moura, A. F. Costa, T. Wendt, R. Sadala and Bromeliário imperialis via Bromeliad Photo Index.

We sampled 76 accessions representing 58 *Vriesea* species, for which we extracted also the molecular data. Such sampling covers the morphological and geographic diversity within genus (Table 1; **see****Supporting Information—Table S1**). To visualize the morphological variation of floral traits in *Vriesea* and test if the species group according to the two different pollination syndromes (hummingbird or bat), we performed a non-metric multidimensional scaling (NMDS; Rabinovitz 1975) using Gower distance (Gower 1971). Non-metric multidimensional scaling is an ordination method which groups similar objects close to one another based on rank distances and has been used for similar kinds of data to build floral morphospaces (Ollerton *et al.* 2009; Chartier *et al.* 2014). In addition to the NMDS, we ran a cluster analysis using the same distance measure to depict morphological relatedness among species. Both analyses were performed in PAST 3.22 (Hammer *et al.* 2001). To test for significant differences among the resulting different clusters, which we expected to correspond to the two pollination syndromes, we ran a PERMANOVA using the R 3.5.0 (R Development Core Team 2018) package ‘vegan’ (Oksanen *et al.* 2018), based on 10 000 permutations. We used data on floral bract colour [mostly yellow tones, Fig. 1E; red tones, Fig. 1B and C; green, Fig. 1I; purple/brown, Fig. 1J; stramineous (with texture and colour of straw, dry and crumbly, Fig. 1G and L)], floral bract size related to flower length (shorter than midpoint of the flower, Fig. 1D; equal to midpoint to longer than flower, Fig. 1C), floral bract imbrication (not imbricate, Fig. 1E; imbricate, Fig. 1B), flower disposition along inflorescence rachis or branches (polystichous, Fig. 1D; distichous, Fig. 1E), time of day of flower anthesis (diurnal; nocturnal), position of flowers at anthesis in relation to floral bract (included with <1/3 of the flower exposed, Fig. 1D; exserted with half of more exposed, Fig. 1J), torsion of the flowers (not-secund, Fig. 1B; partially or totally secund, Fig. 1G, I and L), flower odour (absent; present), corolla colour (mostly yellow, Fig. 1A; white, Fig. 1J; pale-yellow, Fig. 1G; green; wine/purple, Fig. 1H), corolla shape (tubular, Fig. 1A; campanulate, Fig. 1G) and stamens position at anthesis (included, Fig. 1J and L; exserted, Fig. 1A, C and F), **see****Supporting Information—Table S2**. We compiled trait information from the taxonomic and/or phylogenetic works developed by Moura and Costa (2014), Costa *et al.* (2015), Gomes-da-Silva and Souza-Chies (2017), Neves *et al.* (2018) and Uribbe *et al.* (2020). Such works were based on vast sampling of each species both in the field and herbaria collections and therefore we are taking into account the existing intraspecific variation. All traits investigated are potentially involved in attraction and performance (effectiveness, efficiency, efficacy) of pollinators.

**Table 1.** Taxa sampled for chloroplast (*matK* and *rps16-trnK*) and nuclear (*PHYC*) regions, assigned to their respective putative pollination syndrome. Species codes, haplotypes and locality information (country, federal state and municipality). RJ, Rio de Janeiro; ES, Espírito Santo; MG, Minas Gerais; SP, São Paulo; BA, Bahia; PR, Paraná; PE, Pernambuco; SC, Santa Catarina; RS, Rio Grande do Sul.

Pollination syndrome	Species	Species code	Haplotypes cpDNA/*PHYC*	Locality
Outgroup—bats and hawkmoths (Martinelli *et al.* 1994)	*Alcantarea imperialis*	Ai	H1/Hn1	Brazil, RJ, Teresópolis
	*A. regina*	Ar	H2/Hn2	Brazil, RJ, Rio de Janeiro
	*Stigmatodon croceanus*	Scr	H3/Hn3	Brazil, RJ, Santa Maria Madalena
	*S. harrylutheri*	Sha	H4/Hn4	Brazil, ES
	*S. plurifolius*	Spl	H5/Hn5	Brazil, ES
	*S. costae*	Vco	H18/-	Brazil, RJ, Niterói
	*Vriesea (‘Stigmatodon’) oligantha*	Vol	H31/-	Brazil, MG
*Vriesea*—hummingbirds	*V. agostiniana*	Vag	H8/Hn10	Brazil, SP, Caraguatatuba
	*V. amethystina*	Vam	H9/Hn11	Brazil, RJ, Rio de Janeiro
	*V. billbergioides*	Vbi	H11/Hn13	Brazil, RJ, Teresópolis
	*V. botafogensis*	Vbo	H13/Hn15	Brazil, RJ, Rio de Janeiro
	*V. cacuminis*	Vcu	H14/Hn13	Brazil, MG, Ibitipoca
	*V. calimaniana*	Vcl	H15/Hn16	Brazil, ES, Venda Nova do Imigrante
	*V. capixabae*	Vcp	H6/Hn17	Brazil, ES, Ibitirama
	*V. carinata* var. *carinata*	Vcr	H6/Hn18	Brazil, ES, Santa Teresa
	*V. carinata* var. *flavominiata*	Vcrf	H16/Hn19	Brazil, ES, Santa Maria de Jetibá
	*V. carinata* var. *mangaratibensis*	Vcrm	H6/Hn20	Brazil, RJ, Mangaratiba
	*V. duvaliana*	Vdu	H6/Hn22	Brazil, BA, Itacaré
	*V. eltoniana*	Vel	H19/Hn23	Brazil, RJ, Arraial do Cabo
	*V. ensiformis* var. *ensiformis*	Vem	H6/Hn6	Brazil, SP, Cananéia
	*V. ensiformis* var. *ensiformis*	Vem	H20/Hn25	Brazil, PR, Guaraqueçaba
	*V. ensiformis* var. *ensiformis*	Vem	H6/Hn26	Brazil, PE, Caruaru
	*V. ensiformis* var. *ensiformis*	Vem	H20/-	Brazil, SC, Corupá
	*V. ensiformis* var. *bicolor*	Venb	H6/Hn24	Brazil, SP, Caraguatatuba
	*V. erythrodactylon*	Ver	H21/Hn27	Brazil, ES, Santa Teresa
	*V. flammea*	Vfm	H17/Hn29	Brazil, PR, Paranaguá
	*V. flammea*	Vfm	H17/Hn21	Brazil, SP
	*V. flava*	Vfl	H23/Hn30	Brazil, PR, Morretes
	*V. fluviatilis*	Vfu	H25/Hn32	Brazil, RJ, Santa Maria Madalena
	*V. fluviatilis*	Vfu	H19/Hn6	Brazil, RJ, Casimiro de Abreu
	*V. gracilior*	Vgc	H24/-	Brazil, ES, Santa Teresa
	*V. gradata*	Vgr	H26/Hn33	Brazil, RJ, Petrópolis
	*V. gutatta*	Vgu	H27/-	Brazil, SC, Antônio Carlos
	*V. heterostachys*	Vhe	H6/Hn32	Brazil, RJ, Teresópolis
	*V.* aff. *heterostachys*	Vhe	H6/Hn6	Brazil, SP, Ilha do Cardoso
	*V. inflata*	Vif	H6/-	Brazil, SP, São Luis do Paraitinga
	*V.* aff. *inflata*	Vif	H6/Hn7	Brazil, SP, Bananal
	*V. interrogatoria*	Vit	H28/Hn20	Brazil, SP, Cunha
	*V. lubbersii*	Vlu	H47/-	Brazil, RJ, Valença
	*V. maxoniana*	Vma	H48/-	Bolívia, Santa Cruz
	*V. modesta*	Vmo	H6/Hn32	Brazil, RJ, Santa Ma. Madalena
	*V. neoglutinosa*	Vne	H30/Hn36	Brazil, RJ
	*V. paraibica*	Vpa	H32/-	Brazil, RJ, Nova Friburgo
	*V.* aff. *procera*	Vpr	H7/Hn8	Brazil, RS, Torres
	*V. psittacina*	Vpi	H34/Hn6	Brazil, RJ
	*V. recurvata*	Vrc	H35/-	Brazil, BA, Ilhéus
	*V. repandostachys*	Vrp	H6/-	Brazil, ES, Domingos Martins
	*V. rhodostachys*	Vrh	H36/Hn37	Brazil, ES, Santa Teresa
	*V. rhodostachys*	Vrh	H37/Hn38	Brazil, BA, Arataca
	*V. rodigasiana*	Vro	H38/-	Brazil, SP, Cananéia
	*V. rubyae*	Vru	H6/Hn39	Brazil, RJ, Petrópolis
	*V. rubyae*	Vru	H39/Hn40	Brazil, RJ, Angra dos Reis
	*V. sandrae*	Vsa	H40/-	Brazil, BA, Santa Terezinha
	*V. saundersii*	Vau	H43/Hn41	Brazil, RJ, Rio de Janeiro
	*V. scalaris* var. *scalaris*	Vsc	H6/Hn42	Brazil, ES, Santa Teresa
	*V. scalaris* var. *scalaris*	Vsc	H6/Hn42	Brazil, PR, Paranaguá
	*V. scalaris* var. *viridis*	Vscv	H42/Hn42	Brazil, PE, Taquaritinga do Norte
	*V. seideliana*	Vse	H6/Hn6	Brazil, ES, Domingos Martins
	*V. simplex*	Vsi	H6/Hn43	Brazil, ES, Santa Teresa
	*V. simplex*	Vsi	H6/-	Brazil, SP, São Luis do Paraitinga
	*V. sucrei*	Vsu	H44/-	Brazil, RJ, Arraial do Cabo
	*V. sucrei*	Vsu	H44/Hn45	Brazil, ES, Domingos Martins
	*V. taritubensis* var. *brevisepala*	Vtab	H8/-	Brazil, RJ, Guapimirim
	*V. taritubensis* var. *patens*	Vtap	H8/Hn9	Brazil, SP, São Luis do Paraitinga
	*V. taritubensis* var. *taritubensis*	Vta	H8/Hn6	Brazil, RJ, Paraty
	*V. teresopolitana*	Vte	H6/-	Brazil, RJ, Teresópolis
	*V. vagans*	Vva	H6/Hn47	Brazil, SP, Bananal
*Vriesea*—bats	*V. atra*	Vat	H10/Hn12	Brazil, RJ, Teresópolis
	*V. bituminosa*	Vbt	H12/Hn14	Brazil, RJ, Teresópolis
	*V. fenestralis*	Vfe	H22/Hn28	Brazil, RJ, Nova Iguaçu
	*V. fosteriana*	Vfo	H13/Hn31	Brazil, ES, Cachoeiro de Itapemirim
	*V. gigantea*	Vgi	H13/Hn12	Brazil, ES, Domingos Martins
	*V. grandiflora*	Vgd	H13/-	Brazil, RJ
	*V. hydrophora*	Vhy	H13/Hn34	Brazil, RJ, Teresópolis
	*V. longicaulis*	Vlo	H29/Hn35	Brazil, RJ, Teresópolis
	*V. longistaminea*	Vlg	H46/-	Brazil, MG, Mariana
	*V. minuta*	Vmi	H13/-	Brazil, BA
	*V. pabstii*	Vpb	H13/Hn13	Brazil, SP, Bananal
	*V. platynema* var. *variegata*	Vplv	H33/-	Brazil, PR
	*V. pseudatra*	Vps	H13/-	Brazil, RJ, Rio de Janeiro
	*V. sazimae*	Vsz	H41/-	Brazil, RJ, Itatiaia
	*V. sincorana*	Vsc	H43/Hn44	Brazil, BA, Rio de Contas
	*V. unilateralis*	Vun	H45/Hn46	Brazil, SP, Cunha

Lastly, to determine if floral traits predict pollinators and this way validate the pollination syndromes in the genus, we compiled pollinators and floral visitors for 39 *Vriesea* species based on a literature survey and personal observations **[see****Supporting Information—Table S3****]**. We expected to find separate clusters for each syndrome in the ordination analyses described above. Then we tested whether the species with known pollinators fall into their respective syndrome cluster, allowing for confirmation of syndromes.

### Molecular analyses

#### DNA sampling.

We sampled 83 individuals including the 76 accessions of *Vriesea* from the morphological analyses of floral traits and seven outgroup accessions from the sister genera *Alcantarea* and *Stigmatodon* (Table 1; Barfuss *et al.* 2016). We collected young leaves from individuals in natural populations that were preserved in silica gel before DNA extraction using 2× CTAB (Doyle and Doyle 1987) with the modifications of Fay *et al.* (1998). We amplified and sequenced DNA for two chloroplast (*rps16-trnk* and *matK*; Crayn *et al.* 2000; Shaw *et al.* 2007) and one nuclear (*PHYC*; Barfuss *et al.* 2016) regions, as described in Kessous *et al.* (2020). We generated a total of 34 sequences for 21 accessions in this study **[see****Supporting Information—Table S1****]**. Additionally, we included sequences generated by Barfuss *et al.* (2016) and Kessous *et al.* (2020) available in GenBank. For the 16 samples collected by others that were not georeferenced, we estimated geographical coordinates based jointly on locality information from collectors and records from *speciesLink* database (http://splink.cria.org.br/).

#### Haplotype and phylogenetic relationships.

We verified the sequences electropherograms in Chromas 2.33 (Chromas Technelysium, South Brisbane, Australia) and performed multiple sequence alignment in MAFFT 7.0 (Katoh and Standley 2013) following default settings. The final data sets had 83 accessions for the concatenated chloroplast markers with 1543 bp and 60 accessions for the nuclear marker with 664 bp. We analysed chloroplast and nuclear data sets separately to evaluate the evolutionary relationships among haplotypes and taxa.

To first explore genetic variation in *Vriesea*, we inferred the haplotype genealogy for both chloroplast and nuclear data sets. Such analysis can reveal genetic patterns other than that evidenced by the phylogenies, showing how haplotypes group. The use of this analysis is justified to our sampling by the recency of the genus *Vriesea* and the existence of several incipient species still in process of differentiation (Wendt *et al.* 2008; Zanella *et al.* 2016; Neri *et al.* 2017; Kessous *et al.* 2020). Also, such approach has been used in a multispecies context for another bromeliad genera (Krapp *et al.* 2014; Goetze *et al.* 2017). We excluded the mononucleotide repeat length variations due to ambiguous alignment and coded the indels longer than 1 bp as a single mutational event. We identified the haplotypes using DnaSP 5.10.01 (Librado and Rozas 2009) and built the haplotype network using the median-joining method (Bandelt *et al.* 1999) in Network 5 (available at http://www.fluxus-engineering.com).

To infer phylogenetic relationships among chloroplast haplotypes and among species for both chloroplast and nuclear data sets, we performed Bayesian analyses in MrBayes 3.2 (Ronquist *et al.* 2012) using the CIPRES server (Miller *et al.* 2010). We used the HKY nucleotide substitution model calculated in MEGA X (Kumar *et al.* 2018) for each molecular marker separately. Two runs of four Monte Carlo Markov Chain (MCMC) computations were run for 10 000 000 generations. We sampled trees every 1000 generations. The first 25 % of generations were discarded as burn-in. The consensus tree was drawn in FigTree 1.4.2 (Rambaut 2014). We considered well-supported clades those with posterior probabilities (PPs) above 0.95.

#### Genetic divergence.

To assess the consistency of the genetic relationships among taxa, we ran independent principal coordinate analysis (PCoA) for both chloroplast and nuclear data in the R package ‘ape’ (Paradis *et al.* 2004). We used the matrix of pairwise genetic distances amongst the *Vriesea* individuals computed in MEGA under a Maximum Composite Likelihood model, estimating variance using 1000 replicates of bootstrap.

## Results

### Morphological analyses of floral traits—predicting pollinators and validating pollination syndromes

**Figure 2.**
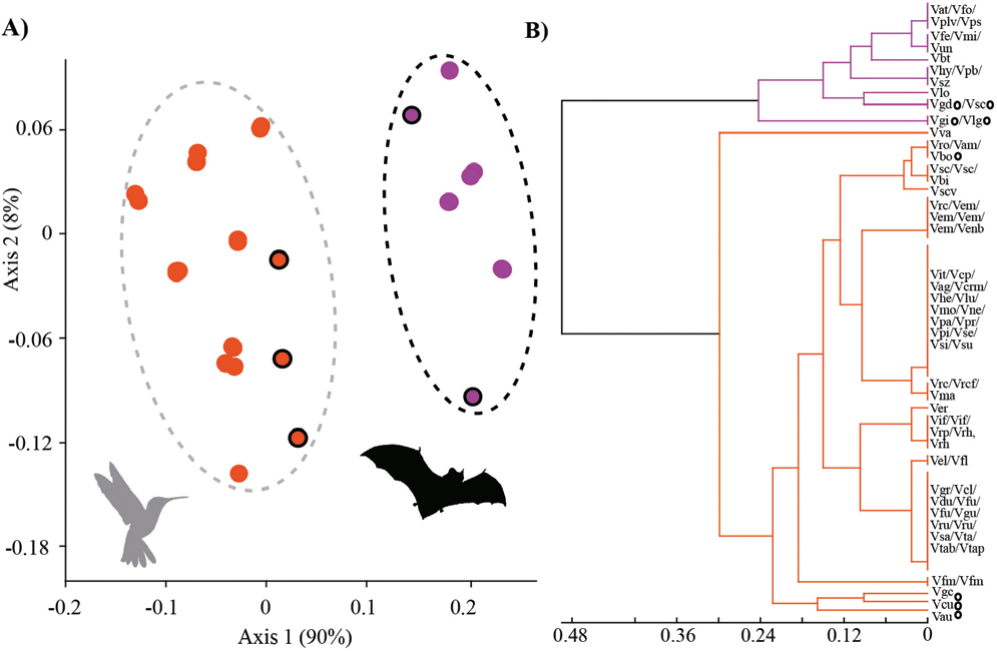
Morphological analyses supporting divergence among *Vriesea* pollination syndromes, hummingbird (orange) and bat (purple). (A) Non-metric multidimensional scaling and (B) cluster dendogram of 11 categorical reproductive characters related to floral bract and flower. Species with morphology distinct from the typical hummingbird- and bat-pollinated flowers (mixed floral traits) are circled in black. Silhouette images of pollinators downloaded from http://phylopic.org.

The NMDS and cluster analysis of floral traits inferred morphological separation of the two pollination syndromes in *Vriesea* (Fig. 2). The first two NMDS axes retained 98 % of the variance of the original data variation (stress 0.14, axis 1 *R*^2^ = 0.90, axis 2 *R*^2^ = 0.08). We found significant differences among clusters corresponding to the different pollination syndromes (*F*(1, 74) = 381.56, *P* < 0.0001). The hummingbird-pollinated species have floral bracts that are mostly red or yellow, frequently equal or longer in length than the flower and are either imbricate or not; flowers are distichous or polystichous, with diurnal anthesis, frequently exserted from floral bracts with half or more exposed, not-secund and odourless; the corolla is yellow or rarely white especially in polystic flowers, is tubular and with mostly exserted stamens (Fig. 1A–F). The bat-pollinated species have floral bracts that are mostly green, purple or brown, frequently equal or longer than the flower, not imbricate; flowers are distichous, with nocturnal anthesis, frequently exserted from the floral bracts with half or more exposed, can be secund (with flowers shifting to an angle of 90° towards one side of the inflorescence) or partially secund (flowers shift <90°), present a garlic odour; corolla is frequently pale-yellow, campanulate, stamens frequently included (Fig. 1G–L).

Species with mixed floral morphology showed intermediate position along NMDS axis 1 (Fig. 2). Such species clearly belong to their respective pollination syndrome cluster but are differentiated from the typical hummingbird (Fig. 1A) and bat (Fig. 1G) flowers. Among hummingbird-pollinated species: *V. cacuminis* has the petal apex erect, often presenting a small flower aperture and included stamens (Fig. 1D); *V. saundersii* presents the same characteristics as the former; additionally, it is highly similar to *V. botafogensis* which has a more typical hummingbird-pollinated flower due to its clearly exserted stamens, but shares overall peculiar morphology of inflorescence and rosette; *V. gracilior* has urceolate flowers with included stamens (Fig. 1E). Among bat-pollinated species: *V. sincorana* (Fig. 1K), *V. gigantea*, *V. grandiflora* and *V. longistaminea* have long exserted stamens that are arranged separately.

We compiled information of pollination biology from the literature for 23 species of the 58 *Vriesea* species sampled here **[see****Supporting Information—Table S3****].** All of the 23 species with known pollinators fell into their respective pollination syndrome cluster, including those with mixed floral traits (Fig. 1D, E and K; **see****Supporting Information—Table S3**). With this result, we showed floral traits to accurately predict pollinators in *Vriesea* confirming pollination syndrome assignments for all species we sampled.

### Haplotype and phylogenetic relationships

The chloroplast data set comprised 1516 bp with a GC (guanine–cytosine) content of 29.9 % and 84 polymorphic sites (51 transitions, 26 transversions and 8 indels) and 48 haplotypes. The nuclear data set was 664 bp with a GC content of 49.1 % and 74 polymorphic sites (39 transitions, 37 transversions and 2 indels) and 47 haplotypes **[see****Supporting Information—Table S1****]**.

**Figure 3.**
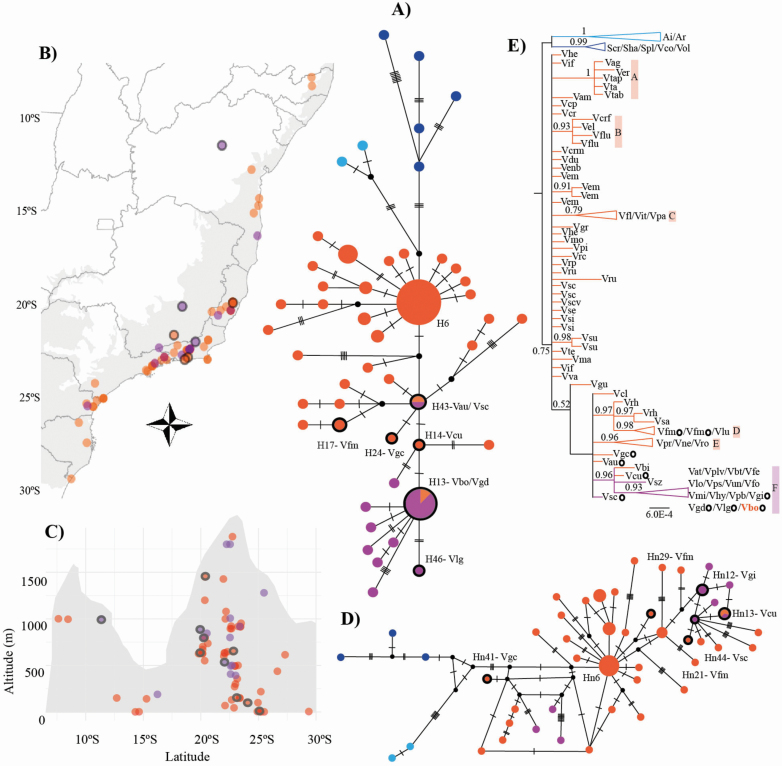
Results based on the cpDNA (*matK* and *rps16-trnK*) and *PHYC* data set for 83 taxa, including 76 *Vriesea* accessions. Coloured symbols represent hummingbird (orange) and bat (purple) syndromes in *Vriesea*. Blue symbols represent the outgroup genera *Alcantarea* and *Stigmatodon*. *Vriesea* species with floral morphology distinct from the typical hummingbird- and bat-pollinated flowers (mixed floral traits) are circled in black. (A) Chloroplast median-joining network showing genetic divergence among pollination groups. Each circle represents a haplotype with the size proportional to its total frequency. We indicate codes for haplotypes of intermediate species and the most frequent H6 and Hn6 (Table 1). Mutational steps are indicated with dashes and hypothetic haplotypes with black dots. (B) Map of *Vriesea* species sampling distribution along the Brazilian Atlantic Forest in grey and (C) across altitude. The mountain profile is illustrative. (D) *PHYC* median-joining network. (E) Chloroplast Bayesian phylogeny with posterior probabilities above 0.50 shown.

The chloroplast haplotype network reflected the different pollination syndromes in *Vriesea* (Fig. 3A) and did not resolve any groupings across latitudinal or elevational space (Fig. 3B and C). *Vriesea* presented two haplogroups pollinated either by hummingbirds and bats, which shared only two haplotypes (H13 and H43, Table 1; Fig. 3A). The haplotypes of each syndrome were resolved in a star-like pattern. The most frequent and central haplotype (H6) was exclusively shared among 21 hummingbird-pollinated species. The second most frequent (H13) was shared among seven bat-pollinated species and the hummingbird-pollinated *V. botafogensis* (Table 1; Fig. 3A; **see****Supporting Information—Fig. S1**). H43 was shared between species of both syndromes, *V. saundersii* and *V. sincorana*. Haplotypes of the species with mixed floral types had an intermediate position in the chloroplast network (Fig. 3A). Haplotypes H6 and H13 were found in individuals occurring from 8°S to 25°S and 16°S to 22°S, at altitudes from the sea level to 1019 m and 150 to 1801 m in the highlands, respectively. Such variation spans almost the entire geographic range of Atlantic Forest *Vriesea* species **[see****Supporting Information—Fig. S4****]**. Most of the remaining plastid haplotypes were restricted to a single species.

The nuclear haplotype network showed weak relationship among species with different pollination syndromes (Fig. 3D). We detected a star-like pattern for the hummingbird syndrome. The most frequent haplotype was shared among six hummingbird-pollinated species (Hn6). Hn13 was shared among species with both pollination syndromes (Table 1; Fig. 3D; **see****Supporting Information—Fig. S1**). The remaining haplotypes were restricted to a single species.

The chloroplast phylogeny inferred six moderately to well-supported clades, despite the backbone of the tree being unresolved (Fig. 3E). The genus was recovered as monophyletic (PP = 0.75) and the hummingbird-pollinated species (clades A, B, C, D and E) were supported (1.00, 0.93, 0.79, 0.98 and 0.96 PP, respectively), while clade F mainly included bat-pollinated species, but also the hummingbird-pollinated *V. billbergioides*, *V. cacuminis* and *V. botafogensis* (PP = 0.93; Table 1). The phylogenies of the chloroplast haplotypes and of the nuclear marker *PHYC* showed low resolution **[see****Supporting Information—Figs S2****and****S3****]**. Finally, the outgroups, *Stigmatodon* and *Alcantarea*, were well-supported as distinct genetic groups in all analyses.

### Genetic divergence

**Figure 4.**
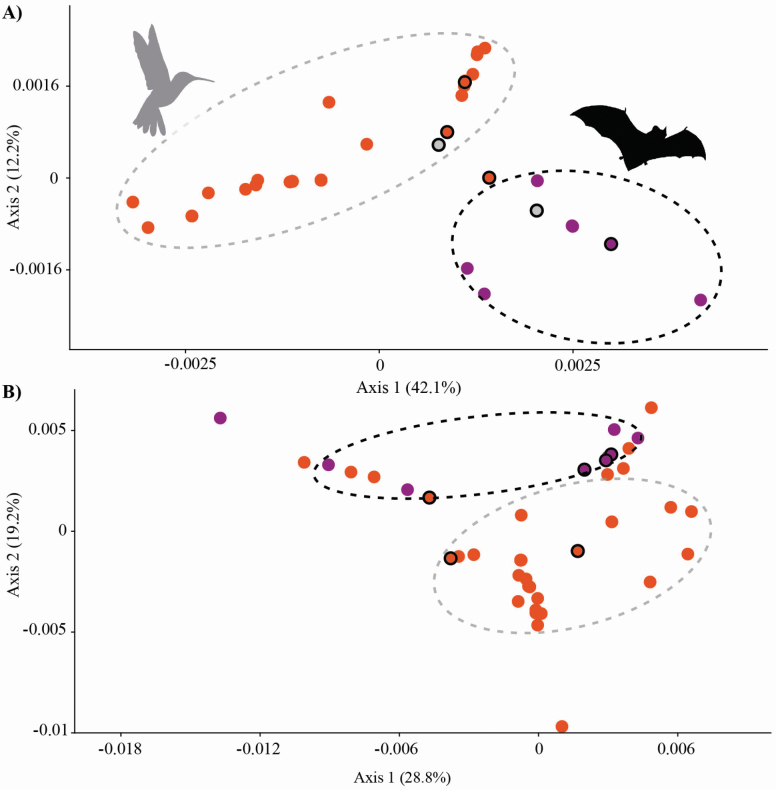
Principal coordinate analysis for (A) cpDNA and (B) *PHYC* data sets showing genetic divergence among hummingbird (orange dots) and bat (purple dots) pollination syndromes in *Vriesea*. Species with morphology distinct from the typical hummingbird- and bat-pollinated flowers (mixed floral traits) are circled in black. Grey dots represent overlap of species from both syndromes which have no genetic divergence. Silhouette images of pollinators downloaded from http://phylopic.org.

The PCoA revealed close genetic relationships of species within the distinct pollination syndromes (Fig. 4). In the chloroplast data, species from both hummingbird and bat pollination syndromes formed distinct genetic clusters (Fig. 4A). For *PHYC*, we detected an overlap among the pollination groups (Fig. 4B). We showed species with mixed floral traits to occupy an intermediate position along axis 1 of chloroplast PCoA plot (Fig. 4A). On the *PHYC* PCoA plot the pattern was diffuse and the species with mixed floral traits were scattered in morphospace, tending to concentrate in the middle of axis 2 (Fig. 4B).

## Discussion

Using morphological and molecular data we show hummingbird and bat pollinators to be closely associated with ecological isolation between *Vriesea* clades (Figs 2 and 3A). The selection imposed by the distinct pollinator groups likely promoted floral traits and species diversification, particularly within clades associated with each pollinator group or derived from shifts among pollinator groups. Also, we confirm floral traits to predict pollinator functional groups in *Vriesea*. We identify species with intermediate position between the different pollination syndrome groupings, which share haplotypes (H13, H43 in Fig. 3A and Hn13 in Fig. 3D), are phylogenetically related (clade F in Fig. 3E) and differ morphologically from the typical hummingbird- and bat-pollinated *Vriesea* flowers by their mixed floral traits (Fig. 1D, E and K). The phylogeny reveals moderately to well-supported clades that are congruent with haplotype relationships and reflect groupings of recognized species complexes (Fig. 3E). We suggest that incipient speciation and incomplete lineage sorting may cause the low genetic divergence detected in the markers surveyed, which also lead to species delimitation challenges in the genus. Future genomic sequencing may further refine the patterns reported here.

### Prediction of pollination syndromes

Considering jointly the literature available for *Vriesea* species pollination biology **[see****Supporting Information—Table S3****]** and our NMDS and clustering results based on 11 categorical traits (Fig. 2), we support floral morphology as a good predictor of functional groups of pollinators. Other studies have shown a similar predictive utility of pollination syndromes in other angiosperm groups (Rosas-Guerrero *et al.* 2014; Serrano-Serrano *et al.* 2017). Here, we focused on the two likely most effective functional groups of pollinators in *Vriesea*: hummingbirds and bats. Although another pollinators such as insects have also been shown to provide pollination services for *Vriesea* species, they are probably less effective as they carry less pollen, potentially drive more self-pollination which can affect seed viability and fly shorter distances than vertebrates (Fleming *et al.* 2009; Schmid *et al.* 2011; Paggi *et al.* 2013). Although a recent study showed hummingbirds as efficient as bees and ants in facilitating pollination per visit in *V. neoglutinosa*, fruit set was reduced when only insects are allowed to visit the flowers, insects potentially bring more self-pollen and seed quality was not examined in order to assess viability (Magalhães *et al.* 2018). Additionally, *Vriesea* flowers produce large volume of nectar which results in higher amount of sugar, reinforcing the strong association with vertebrate pollinators whose energetic needs are higher than insects (Sazima *et al.* 1995; Göttlinger *et al.* 2019). Some cases of bat-pollinated flowers have been reported to be visited by hummingbirds at dawn, but when hummingbirds feed on the small amount of nectar left by bats in withered flowers, it does not compete with the performance of primary pollinators (Sazima *et al.* 1995; Aguilar-Rodríguez *et al.* 2019).

The morphological characters analysed here are fundamental not only to define syndromes but also to define pollinator performance. Bergamo *et al.* (2019) showed that in long tubular flowers, floral bracts create colour contrast and enhance the petal signal more for hummingbirds than for bees, hence creating an avoidance mechanism against nectar robbing bees. We measured floral bract size related to flower length and its relative position, traits that are directly linked to bract/petals contrast. We also measured flower position and torsion following the hypothesis that in species with distichous and secund flowers, pollinators can easily reach a greater number of flowers since they all face the same side (Costa *et al.* 2014; Aguilar-Rodríguez *et al.* 2019). Flower torsion is especially important for bats when pollinating compound inflorescences, since the flowers from each branch are properly exposed. Further, stamen position influences pollinator effectiveness due to the placement of pollen deposition along the pollinator body. Rocca and Sazima (2013) showed that short-billed hummingbirds receive pollen on the top of the head and deposit it on the centre of the stigma, whereas long-billed species receive pollen on the proximal part of the bill and deposit it on the lower lobe of the stigma in *V. rodigasiana*. In this case, short-billed hummingbird-pollinated flowers had six times more pollen tubes formed than long-billed ones. In bat-pollinated species, the pollen of flowers with anthers located in the lower side of corolla, such as in *V. bituminosa*, is concentrated on bat chins, whereas the spread anthers of *V. gigantea* deposit pollen all over the face of bats (Sazima *et al.* 1995).

### Ecological isolation drives genetic structure in *Vriesea*

Our results suggest an important role of ecological instead of geographical isolation in driving genetic structure in *Vriesea* (Fig. 3). The specialized association with different vertebrate pollinator groups has resulted in ecological isolation amongst clades within *Vriesea*. We found few shared haplotypes between syndromes in our chloroplast and nuclear data (Fig. 3A and D) and demonstrated clear floral specialization on both hummingbirds and bats for pollination (Fig. 1). It has been argued that strong plant–pollinator interactions lead to an increase in species diversity (Givnish *et al.* 2014; Lagomarsino *et al*. 2016; Serrano-Serrano *et al.* 2017). We hypothesize that the association with hummingbirds and bats is a main biotic driver of *Vriesea* diversification. In addition, pollinator shifts can open new adaptive space for species diversification and distinct mechanisms may increase speciation within each main pollinator group (which seems to be where most diversification in the genus occurs), such as (i) floral specialization on specific pollinator species; (ii) efficiency of pollen transfer and deposition by specific pollinator species which affect connectivity among populations resulting in allopatric speciation; (iii) different flowering time and pollinator behaviour when foraging; and (iv) different pollinator species distribution along altitude and habitat types according to their physiological preferences (Aguilar-Rodriguez *et al.* 2019; Kessler *et al.* 2020).

We found stronger structure for syndromes in the chloroplast DNA compared to the more diffuse pattern for the nuclear markers, and a proportionally low number of haplotypes for the chloroplast than nuclear data (Fig. 3A and D; Table 1). Maternally inherited markers such as those from the chloroplast are generally highly structured and present low variation (Petit *et al.* 2005; Goetze *et al.* 2017), at least when compared to nuclear DNA (Barfuss *et al.* 2016). Despite the high structure, haplotype sharing does occur within and among syndromes, as well as among species collected in distant localities, for both chloroplast and nuclear DNA, albeit infrequently. For example, haplotypes H6 and Hn12 are found in species occurring along a range of ca. 3000 km (Table 1; **see****Supporting Information—Table S1**). Such findings may reflect incipient speciation due to recent species diversification within the genus (crown age 4–2 Mya, Kessous *et al.* 2020), preventing sufficient time to accumulate genetic differences and causing various species complexes. With this, our findings suggest incomplete lineage sorting through the retention of ancestral polymorphisms. Incomplete lineage sorting often explains such disperse spatial patterns of shared genetic variation across species (Goetze *et al.* 2017). Also, interspecific gene flow has been shown by previous studies to occur in *Vriesea* (Zanella *et al.* 2016; Neri *et al.* 2017), as well as in its sister group *Alcantarea* (Lexer *et al.* 2016).

We did not detect a geographic pattern from our data (like shown by Krapp *et al.* (2014) for other bromeliad genera) and show that *Vriesea* species from each syndrome are widely distributed across latitude and altitude in the Atlantic Forest (Fig. 3B and C; **see****Supporting Information—Fig. S4**). Studies comparing hummingbird- and bat-pollinated plant assemblages in the Atlantic Forest at different altitudinal ranges showed higher species diversity of both *Vriesea* and pollinators in the lowlands (Sazima *et al.* 1999; Buzato *et al.* 2000). For bromeliads in general, it has been argued that bat-pollinated species are more diverse at humid mid-elevations and lowlands, whereas hummingbird-pollinated species are more diverse in mid-elevation to highlands, which coincides with the physiological demands of the different groups of pollinators (see review of Kessler *et al.* 2020). In addition, these authors discuss the shifts among pollination syndromes to occur predominantly in transition zones at mid-elevation areas. Such distribution pattern does not agree with the one of the intermediate species we recognize in this study, as they are distributed along whole range of *Vriesea* species (Fig. 3C). Considering the high species richness of *Vriesea*, studies on pollination biology to unveil such patterns remain scarce.

Regardless of whether there are distributional differences across altitude between the two syndromes, there are certainly differences in habitat types. Bat-pollinated *Vriesea* usually occur in open habitats as epiphytes in the forest canopies, or are rupiculous or saxicolous in the highlands, making it easier for bats to echolocalize the flowers in addition to the olfactory and visual attractants (Gonzalez-Terrazas *et al.* 2016), whereas hummingbird-pollinated species are more frequent epiphytes in the forest understory, in both highlands and lowlands, or terrestrial in the restingas (for occurrence and habitat information on *Vriesea* species, see Flora do Brasil 2020, continuously updated). The interaction of multiple biotic and abiotic factors drives diversification in species-rich Neotropical clades (Antonelli *et al.* 2018). As a first step to understand *Vriesea* diversification we here show a strong signal of pollinators in shaping genetic structure and likely contributing to its high species diversity.

### Infrageneric relationships

We recovered some well-supported clades consisting exclusively of hummingbird-pollinated species and a large clade including mostly bat-pollinated species, but also the species with mixed morphological traits, despite the overall low resolution of the phylogeny (Fig. 3E). Additionally, these well-supported clades comprise species complexes. Clade A includes species of *V. incurvata* complex (Neves *et al.* 2018); clade B together with clade C, which is formed by species of *V. paraibica* group (Costa *et al.* 2009), comprise the ‘inflated group’. The ‘inflated group’ was recovered in the phylogeny produced by Gomes-da-Silva and Souza-Chies (2017), but without including *V. fluviatilis* (former *V. gradata* var. *bicolor*, Kessous and Costa 2017). The monophyly of *V. corcovadensis* group was also inferred (Gomes-da-Silva and Souza-Chies 2017; Machado *et al.* 2020) and we corroborate it here (clade D). Clade E includes species of the *V. procera* complex Uribbe *et al.* (2020). Clade F mainly includes *Vriesea* with bat pollination syndrome but also the mixed forms: the hummingbird-pollinated *V. cacuminis* and *V. botafogensis*, and the bat-pollinated *V. gigantea*, *V. grandiflora*, *V. longistaminea* and *V. sincorana* (Table 1; Fig. 3E). Differences mainly consist in the corolla aperture, shape and position of stamens at anthesis (see Fig. 1D, E and K; Costa *et al.* 2015).

Sanmartin-Gajardo and Sazima (2005) identified a putative transition from hummingbird to bat pollination in a species of Sinningieae (Gesneriaceae) with intermediate floral traits. Floral specialization on hummingbird syndromes may not be an evolutionary dead end, as transitions may occur to hawkmoth or bat, for example (Tripp and Manos 2008) and even the reverse, from bat to hummingbird (Lagomarsino *et al.* 2017). For bromeliads, Aguilar-Rodriguez *et al.* (2019) showed pollination by hummingbirds to be the ancestral syndrome, with bat pollination originating multiple times. The authors sampled only three *Vriesea* species (*sensu*Barfuss *et al.* 2016), but identified the genus as one of the most representative among the chiropterophilous in Bromeliaceae. Kessler *et al.* (2020) reported three shifts from hummingbird to bat and one shift from bat to hummingbird in *Vriesea* (*sensu*Gomes-da-Silva and Souza-Chies 2017). To properly track these shifts in pollination syndromes in *Vriesea*, a robust phylogeny resolved at the shallow phylogenetic relationships is needed.

We suggest that species with mixed floral traits are a window into shifts between pollinator syndrome, constituting possible transitional floral types in *Vriesea*. Interestingly, despite the morphological differences, the species with mixed floral types clearly belong to their respective syndrome and may be a product of transitions as well as reversions. Species that are product of reversions to the ancestral pollination syndromes likely do not show the exact original floral traits, only their general appearance (Tripp and Manos 2008). Such transitions and reversions have a historical baggage and may likely be driven by selection pressures imposed by pollinators.

## Concluding Remarks

Our results support the hypothesis that pollinators drive ecological isolation in *Vriesea* bromeliads, which show clear floral specialization towards hummingbirds and bats. In our assessment, we defined floral trait diversity and identified possible transitional floral types in *Vriesea*, generating insights on shifts between pollination syndromes to be further explored. Also, we demonstrated the utility of pollination syndromes in predicting functional pollinator groups in *Vriesea*. The morphological-genetic continuum we identified here may be typical of ongoing pollinator-driven speciation. Unveiling the agents and mechanisms behind the evolution of species complexes in biodiversity hotspots such as the Atlantic Forest is of high relevance to further understand the evolution of plants.

## Supporting Information

The following additional information is available in the online version of this article—

**Table S1.** Taxa sampled for chloroplast (*rps16-trnK* and *matK*) and nuclear (*PHYC*) regions, with geographical coordinates, altitude, voucher information and GenBank accession numbers.

**Table S2.** Floral traits measured for the morphological analyses.

**Table S3.** Known pollinators and floral visitors of *Vriesea* species.

**Figure S1.** cpDNA (*matK* and *rps16-trnK*) and *PHYC* median-joining networks showing genetic divergence among *Vriesea* pollination groups.

**Figure S2.** Bayesian phylogeny of cpDNA haplotypes (*matK* and *rps16-trnK*) for 83 taxa, including 76 *Vriesea* accessions.

**Figure S3.** Bayesian phylogeny based on *PHYC* data set for 60 taxa, including 55 *Vriesea* accessions.

**Figure S4.** Map of Brazil with geographic distribution of (A) hummingbird- (orange) and (B) bat-pollinated (purple) *Vriesea* species along the Atlantic Forest (in grey).

plaa046_suppl_Supplementary_Material

plaa046_suppl_Supplementary_Table_S2

## Data Availability

All data are provided in **Supporting Information** and available at GenBank https://www.ncbi.nlm.nih.gov/genbank/.
