# Supplementary material for: Pollinators drive floral evolution in an Atlantic Forest genus
Source: AoB Plants. 2020 Aug 22;12(5):plaa046. doi: 10.1093/aobpla/plaa046 (PMC7532729; doi:10.1093/aobpla/plaa046)
Supplement: plaa046_suppl_Supplementary_Material [file plaa046_suppl_supplementary_material.pdf]

Pollinators drive floral evolution in an Atlantic Forest genus

Beatriz Neves, Igor M. Kessous, Ricardo L. Moura, Dayvid R. Couto, Camila M. Zanella,
Alexandre Antonelli, Christine D. Bacon, Fabiano Salgueiro and Andrea F. Costa

Supporting Information

Table S1. Taxa sampled for chloroplast (*rps16-trnK* and *matK*) and nuclear (*PHYC*) regions, assigned to their respective pollination syndrome. Geographical coordinates, altitude, voucher information and Genbank accession are given.

**Pollination syndrome**	**Species**	**Geographical coordinate Latitude / Longitude**		**Altitude (m)**	**Voucher**	**GeneBank accession** (*rps16-trnK, matK, PHYC*)
Outgroup- bats and hawkmoths	*Alcantarea imperialis*	-22.4603	-42.99556	1246	Kessous 178 (R)	MN203393, MN203299, MN203223
	A. regina	-22.9536	-43.16333	35	B100171454	MN203395, KX754111, KX753978
	Stigmatodon croceanus	-21.9508	-41.94722	810	Leme 4316 (HB)	MN203417, KX754200, KX754067
	S. harrylutheri	-	-	-	Leme 8026 (HB)	MN203422, KX754219, KX754086
	S. plurifolius	-	-	-	Araújo s.n. (HB72850)	MN203424, KX754154, KX754021
	S. costae	-	-	-	Silva 115 (RB)	MN20341, MN203311, -
	Vriesea ('Stigmatodon') oligantha	-	-	-	Couto 3127 (R)	MN20347, MN203366, -

Vriesea-hummingbirds	*V. agostiniana*	-23.5714	-45.49222	150	Kessous 154 & Pinto (R)	MN203434, MN203325, MN203245
	V. amethystina	-22.9403	-43.28917	500	Ribeiro s.n. (R1999085)	MN203435, MN203326, MT874418
	V. billbergioides	-22.4533	-43.02	1883	Couto 3069 (R)	MT874420, MT874385, MT874405
	V. botafogensis	-22.9719	-43.02556	150	França 25(R)	MT874421, MT874386, MT874406
	V. cacuminis	-21.6958	-43.89666	1428	Couto 2819 (R)	MN203440, MN203331, MN203248
	V. calimaniana	-20.3683	-41.2439	1450	Neves 354 (R)	MT874422, MT874387, MT874407
	V. capixabae	-20.445	-41.73611	736	Venda16 (RBvb)	MN203441, MN203332, MN203249
	V. carinata var. *carinata*	-19.9669	-40.53472	671	Neves 294 (R)	MT874423, MT874388, MT874408
	V. carinata var. *flavominiata*	-20.1767	-40.91861	1200	Neves 304 (R)	MN203444, MN203335, MN203252
	V. carinata var. *mangaratibensis*	-22.6292	-43.8981	130	Leme 656 (HB)	MN203445, MN203336, MN203253
	V. duvaliana	-14.3861	-39.03528	1	Neves 278 (R)	MN203447, MN203339, MN203256
	V. eltoniana	-22.9942	-41.98694	300	Neves186 (R)	MN203448, MN203340, MN203257
	V. ensiformis var. *ensiformis*	-25.0747	-47.92472	5	Kessous 164 & Pinto (R)	MN203450, MN203342, MN203259
	V. ensiformis var. *ensiformis*	-25.1911	-48.29972	100	Kessous 240 (R)	MT874424, MT874389, MT874409
	V. ensiformis var. *ensiformis*	-8.37278	-36.02556	996	Kessous 79 (R)	MT874425, MT874390, MT874410
	V. ensiformis var. *ensiformis*	-26.3706	-49.32028	437	Kessous 247(R)	MT874426, MT874391, -
	V. ensiformis var. *bicolor*	-23.62	-45.41	150	Kessous 155 & Pinto (R)	MN203449, MN203341, MN203258
	V. erythrodactylon	-19.9669	-40.53472	702	Neves 296 (R)	MN203451, MN203343, MN203260

V. flammea	-25.52	-48.5092	1	Kessous 238 & Neves (R)	MN203453, MN203345, MN203262
V. flammea	-24.099	-48.365	103	RB560880	MN203446, MN203337, MN203254
V. flava	-25.3597	-48.88389	218	Kessous 245 & Neves (R)	MN203454, MN203346, MN203263
V. fluviatilis	-21.9575	-41.94694	634	Kessous103 (R)	MT874427, MT874392, MT874411
V. fluviatilis	-22.3153	-42.19028	69	Kessous115 (R)	MT874428, MT874393, MT874412
V. gracilior	-19.9711	-40.53056	627	Kessous 63 (R)	MN203457, MN203348, -
V. gradata	-22.4781	-43.14667	895	Kessous 167 (R)	MN203459, MN203351, MN203267
V. gutatta	-27.45	-48.87	614	Martinelli 14921 (RB)	MN203461, MN203353, -
V. heterostachys	-22.4603	-42.99556	1079	Kessous 120 & Pinto (R)	MN203462, MN203354, MN203268
V. aff. *heterostachys*	-25.0747	-47.92472	5	Kessous 165 (R)	MT874419, MT874384, MT874404
V. inflata	-23.3558	-45.11806	915	Neves 377 (R)	MN203464, MN203357, MN203271
V. aff. *inflata*	-22.8739	-44.44861	494	Neves 314 & Kessous (R)	MN203431, MN203322, MN203242
V. interrogatoria	-23.1989	-44.98944	919	Kessous 218 & Neves (R)	MN203465, MN203358, MN203272
V. lubbersi	-22.2456	-43.7003	600	RBvb469	MN203469, MN203362, MN203275
V. maxoniana	-	-	-	Krömer 5597 (HEID)	MN203470, KX754169, KX754036
V. modesta	-21.9508	-41.94722	634	Neves 343 (R)	MN203472, MN203364, MN203277
V. neoglutinosa	-	-	-	Baumgratz 35 (RB)	MN203473, MN203365, MN203278
V. paraibica	-22.505	-43.1786	922	Uribbe 260 (R)	MN203476, MN203368, MN203280

V. aff. *procera*	-29.3756	-49.75694	5	Kessous 246 & Neves (R)	MN203433, MN203324, MN203244
V. psittacina	-	-	-	Barbosa 162 (RB)	MN203478, MN203371, MN203283
V. recurvata	-14.7761	-39.08972	2	Kessous 212 (R)	MN203479, MN203371, -
V. repandostachys	-20.3749	-40.65932	551	Neves 349 (R)	MN203479, MN203373, -
V. rhodostachys	-19.9111	-40.55278	627	Neves 299 (R)	MT874430, MT874395, MT874413
V. rhodostachys	-15.1708	-39.34972	141	Leitman 268 (RB)	MT874431, MT874396, MT874414
V. rodigasiana	-25.0747	-47.92472	5	Moura 1013 (R)	MN203481, MN203374, -
V. rubyae	-22.5286	-43.13527	895	Neves 331 (R)	MN203483, MN203376, MN203284
V. rubyae	-23.0067	-44.3181	272	Moura 973 (R)	MT874432, MT874397, MT874415
V. sandrae	-12.8453	-39.47528	150	Neves 272 (R)	MT874433, MT874398, -
V. saundersii	-23.0008	-43.28472	648	WU4316	MN203485, AY614036, KX753894
V. scalaris var. *scalaris*	-19.9111	-40.55278	627	Kessous 142 (R)	MT874434, MT874399, MT874416
V. scalaris var. *scalaris*	-25.52	-48.5092	2	Kessous 239 & Neves (R)	MN203487, MN203379, MN203286
V. scalaris var. *viridis*	-7.90306	-36.0442	1001	Kessous 189 (R)	MN203488, MN203380, MN203287
V. seideliana	-20.3756	-40.65922	800	Neves 348 (R)	MN203489, MN203381, MN203288
V. simplex	-19.9111	-40.55278	627	Kessous 137 (R)	MN203490, MN203382, MN203289
V. simplex	-23.3383	-45.15	915	Neves 368 (R)	MT874435, MT874400, -
V. sucrei	-22.9956	-41.98611	356	Neves 188 (R)	MN203492, MN203384, MN203292

	V. sucrei	-20.3756	-40.65922	800	Neves 351 (R)	MT874436, MT874401, -
	V. taritubensis var. *brevisepala*	-22.5153	-43	646	Neves 40 (R)	MT874437, MT874402, -
	V. taritubensis var. *patens*	-23.3383	-45.15	953	Neves 191 (R)	MN203477, MN203369, MN203294
	V. taritubensis var. *taritubensis*	-23.0258	-44.61306	45	Neves 218 (R)	MN203493, MN203385, MN203293
	V. teresopolitana	-22.4122	-42.9656	1000	Neves 385 (R)	MN203494, MN203386, MN203291
	V. vagans	-22.8739	-44.44861	494	Kessous 225 (R)	MT874438, MT874403, MT874417
Vriesea- bats	*V. atra*	-22.4122	-42.9656	1007	Moura 941 (R)	MN203437, MN203328, MN203246
	V. bituminosa	-22.461	-43.02172	800	Kessous 179 (R)	MN203438, MN203329, MN203247
	V. fenestralis	-22.7592	-43.4511	393	Lima 6485 (RB)	MN203452, MN203344, MN203261
	V. fosteriana	-20.6339	-41.38139	845	Moura 861 (R)	MN203455, MN203347, MN203264
	V. gigantea	-20.3633	-40.6592	800	Martinelli 15646 (RB)	MN203456, MN203348, MN203265
	V. grandiflora	-22.0497	-42.6747	528	Moura 871 (R)	MN203460, MN203352, -
	V. hydrophora	-22.4492	-43.00306	1801	Couto 3072 (R)	MN203463, MN203355, MN203269
	V. longicaulis	-22.4575	-43.02417	1801	Couto 3071 (R)	MN203467, MN203360, MN203274
	V. longistaminea	-20.16	-43.42389	879	RBvb975	MN203468, MN203361, MN203275
	V. minuta	-16.3833	-39.13333	189	Moura 829 (R)	MN203471, MN203363, -
	V. pabstii	-22.8739	-44.44861	494	Kessous 229 (R)	MN203475, MN203367, MN203279
	V. platynema var. *variegata*	-25.4769	-48.8344	1280	Kowalski 29 (HUPG)	MN203496, MN203389, -

V. pseudatra	-22.9028	-43.2075	500	Moura 580 (R)	MT874429, MT874394, -
V. sazimae	-22.3688	-44.7492	407	Moura 1163 (R)	MN203486, MN203378, MN203285
V. sincorana	-11.55	-41.1561	800	Moura 864 (R)	MN203491, MN203383, MN203290
V. unilateralis	-23.1989	-44.98944	919	Kessous & Neves 230 (R)	MN203495, MN203388, MN203295

Table S2. Floral traits measured for the Correspondence Analysis: floral bract color (mostly 0 = yellow tones, 1 = red tones, 2 = green, 3 = purple/brown, 4 = stramineous), floral bract size related to flower lenght (0 = shorter than midpoint of the flower, 1 = equal to midpoint to longer than flower), floral bract imbrication (0 = not imbricate, 1 = imbricate), flower disposition along inflorescence rachis or branches (0 = polystichous, 1 = distichous), time of day of flower anthesis (0 = diurnal, 1 = nocturnal), position of flowers at anthesis in relation to floral bract (0 = included with less than 1/3 of the flower exposed, 1 = exserted with half of more exposed), torsion of the flowers (0 = not-secund, 1 = partially or totally secund), flower odor (0 = absent, 1 = present), corolla color (mostly 0 = yellow, 1 = white, 2 = pale-yellow, 3 = green, 4 = wine/purple), corolla shape (0 = tubular, 1 = campanulate), stamens position at anthesis (0 = included, 1 = exserted).

Pollination syndrome	Species	Floral bract color	Floral bract size related to flower lenght	Floral bract imbrication	Flower disposition along rachis	Time of flower anthesis	Position of flowers at anthesis in relation to floral bract	Torsion of the flowers	Flower odor	Corolla color	Corolla shape	Stamens position at anthesis
Vriesea - hummingbirds	*Vriesea agostiniana*	1	1	0	1	0	1	0	0	0	0	1
	V. amethystina	0	0	0	1	0	1	0	0	0	0	1
	V. billbergioides	1	0	0	1	0	1	0	0	0	0	1
	V. botafogensis	0	0	0	1	0	1	0	0	0	0	1
	V. cacuminis	1	0	0	1	0	1	0	0	0	0	0
	V. calimaniana	1	1	1	1	0	1	0	0	0	0	1
	V. capixabae	1	1	0	1	0	1	0	0	0	0	1

Pollination syndrome	Species	Floral bract color	Floral bract size related to flower lenght	Floral bract imbrication	Flower disposition along rachis	Time of flower anthesis	Position of flowers at anthesis in relation to floral bract	Torsion of the flowers	Flower odor	Corolla color	Corolla shape	Stamens position at anthesis
	V. carinata	0	1	0	1	0	1	0	0	0	0	1
	V. carinata var. *flavominiata*	0	1	0	1	0	1	0	0	0	0	1
	V. carinata var. *mangaratibensis*	1	1	0	1	0	1	0	0	0	0	1
	V. duvaliana	1	1	1	1	0	1	0	0	0	0	1
	V. eltoniana	0	1	1	1	0	1	0	0	0	0	1
	V. ensiformis var. *ensiformis*	1	1	0	1	0	0	0	0	0	0	1
	V. ensiformis var. *ensiformis*	1	1	0	1	0	0	0	0	0	0	1
	V. ensiformis var. *ensiformis*	1	1	0	1	0	0	0	0	0	0	1
	V. ensiformis var. *ensiformis*	1	1	0	1	0	0	0	0	0	0	1
	V. ensiformis var. striata	1	1	0	1	0	0	0	0	0	0	1
	V. erythrodactylon	0	1	1	1	0	0	0	0	0	0	1
	V. flammea	1	1	0	2	0	1	0	0	1	0	1
	V. flammea	1	1	0	2	0	1	0	0	1	0	1

Pollination syndrome	Species	Floral bract color	Floral bract size related to flower lenght	Floral bract imbrication	Flower disposition along rachis	Time of flower anthesis	Position of flowers at anthesis in relation to floral bract	Torsion of the flowers	Flower odor	Corolla color	Corolla shape	Stamens position at anthesis
	V. flava	0	1	1	1	0	1	0	0	0	0	1
	V. fluviatilis	1	1	1	1	0	1	0	0	0	0	1
	V. fluviatilis	1	1	1	1	0	1	0	0	0	0	1
	V. gracilior	1	1	0	1	0	1	0	0	0	0	0
	V. gradata	1	1	1	1	0	1	0	0	0	0	1
	V. gutatta	1	1	1	1	0	1	0	0	0	0	1
	V. heterostachys	1	1	0	1	0	1	0	0	0	0	1
	V. aff. *heterostachys*	1	1	0	1	0	1	0	0	0	0	1
	V. inflata	1	1	1	1	0	0	0	0	0	0	1
	V. aff. *inflata*	1	1	1	1	0	0	0	0	0	0	1
	V. interrogatoria	1	1	0	1	0	1	0	0	0	0	1
	V. lubbersi	1	1	0	1	0	1	0	0	0	0	1
	V. maxoniana	0	1	0	1	0	1	0	0	0	0	1

Pollination syndrome	Species	Floral bract color	Floral bract size related to flower lenght	Floral bract imbrication	Flower disposition along rachis	Time of flower anthesis	Position of flowers at anthesis in relation to floral bract	Torsion of the flowers	Flower odor	Corolla color	Corolla shape	Stamens position at anthesis
	V. modesta	1	1	0	1	0	1	0	0	0	0	1
	V. neoglutinosa	1	1	0	1	0	1	0	0	0	0	1
	V. paraibica	1	1	0	1	0	1	0	0	0	0	1
	V. aff. *procera*	1	1	0	1	0	1	0	0	0	0	1
	V. psittacina	1	1	0	1	0	1	0	0	0	0	1
	V. recurvata	1	1	0	1	0	0	0	0	0	0	1
	V. repandostachys	1	1	1	1	0	0	0	0	0	0	1
	V. rhodostachys	1	1	1	1	0	0	0	0	0	0	1
	V. rhodostachys	1	1	1	1	0	0	0	0	0	0	1
	V. rodigasiana	0	0	0	1	0	1	0	0	0	0	1
	V. rubyae	1	1	1	1	0	1	0	0	0	0	1
	V. rubyae	1	1	1	1	0	1	0	0	0	0	1
	V. saundersii	0	1	0	1	0	1	1	0	0	0	0

Pollination syndrome	Species	Floral bract color	Floral bract size related to flower lenght	Floral bract imbrication	Flower disposition along rachis	Time of flower anthesis	Position of flowers at anthesis in relation to floral bract	Torsion of the flowers	Flower odor	Corolla color	Corolla shape	Stamens position at anthesis
	V. sandrae	1	1	1	1	0	1	0	0	0	0	1
	V. scalaris var. *scalaris*	1	0	0	1	0	1	0	0	0	0	1
	V. scalaris var. *scalaris*	1	0	0	1	0	1	0	0	0	0	1
	V. scalaris var. viridis	2	0	0	1	0	1	0	0	0	0	1
	V. seideliana	1	1	0	1	0	1	0	0	0	0	1
	V. simplex	1	1	0	1	0	1	0	0	0	0	1
	V. simplex	1	1	0	1	0	1	0	0	0	0	1
	V. sucrei	1	1	0	1	0	1	0	0	0	0	1
	V. sucrei	1	1	0	1	0	1	0	0	0	0	1
	V. taritubensis var. brevisepala	1	1	1	1	0	1	0	0	0	0	1
	V. taritubensis var. *patens*	1	1	1	1	0	1	0	0	0	0	1
	V. taritubensis var. *taritubensis*	1	1	1	1	0	1	0	0	0	0	1
	V. teresopolitana	1	1	1	1	0	1	0	0	0	0	1

Pollination syndrome	Species	Floral bract color	Floral bract size related to flower lenght	Floral bract imbrication	Flower disposition along rachis	Time of flower anthesis	Position of flowers at anthesis in relation to floral bract	Torsion of the flowers	Flower odor	Corolla color	Corolla shape	Stamens position at anthesis
	V. vagans	4	0	0	1	0	1	1	0	0	0	1
Vriesea- bats	*V. atra*	3	1	0	1	1	1	0	1	2	1	0
	V. bituminosa	1-2	1	0	1	1	1	0	1	4	1	0
	V. fenestralis	2	1	0	1	1	1	0	1	2	1	0
	V. fosteriana	3	1	0	1	1	1	0	1	2	1	0
	V. gigantea	2	0	0	1	1	1	1	1	2	1	1
	V. grandiflora	3	1	0	1	1	1	0	1	2	1	1
	V. hydrophora	2	1	0	1	1	1	1	1	2	1	0
	V. longicaulis	3	1	0	1	1	1	1	1	2	1	1
	V. longistaminea	2	0	0	1	1	1	1	1	2	1	1
	V. minuta	2	1	0	1	1	1	0	1	2	1	0
	V. pabstii	2	1	0	1	1	1	1	1	2	1	0
	V. platynema var. *variegata*	3	1	0	1	1	1	0	1	2	1	0

Pollination syndrome	Species	Floral bract color	Floral bract size related to flower lenght	Floral bract imbrication	Flower disposition along rachis	Time of flower anthesis	Position of flowers at anthesis in relation to floral bract	Torsion of the flowers	Flower odor	Corolla color	Corolla shape	Stamens position at anthesis
	V. pseudatra	3	1	0	1	1	1	0	1	2	1	0
	V. sazimae	2	1	0	1	1	1	1	1	2	1	0
	V. sincorana	3	1	0	1	1	1	0	1	2	1	1
	V. unilateralis	2	1	0	1	1	1	0	1	2	1	0

See also .csv file.

Table S3. Known pollinators and floral visitors of *Vriesea* species in Brazil and the domains in which species occur (AF: Atlantic Forest and CE: Cerrado). Species sampled in this study are marked in bold. As a first assessment, we here report all studies we found mentioning pollinators or visitors of *Vriesea* species, regardless the methodology applied. Symbols: *indicates visitors, §*V. morreniana* is an artificial hybrid and does not exist in nature, based on the locality information the species is probably *V. interrogatoria* L. B. Sm. (Costa, 1997).

Vriesea species	Brazilian domain	Pollinator / Visitor	Pollinator / Visitor species	Reference	Details of referenced studies
V. altodaserrae	AF	Hummingbirds	*Amazilia versicolor, Anthracothorax nigricollis, Clytolaema rubricauda, Florisuga fusca, Leucochloris albicollis, Lophornis magnificus, Phaethornis eurynome, Thalurania glaucopis, Melanotrochilus fuscus, Chlorostilbon aureoventris (= Chlorostilbon lucidus)*	Kaehler *et al.* (2005), Machado and Semir (2006), Nunes *et al.* (2018)	*A. versicolor, A. nigricollis, C. rubricauda, L. albicollis, L. magnificus, P. eurynome, T. glaucopis* pollinated flowers touching their reproductive parts (Kaehler *et al.* 2005). *A. versicolor, A. nigricollis, C. rubricauda, L. albicollis, P. eurynome, T. glaucopis, M. fuscus, C. aureoventris* (= *C. lucidus*) pollinated flowers touching their reproductive parts (Machado and Semir 2006).
V. altomacaensis	AF	Hummingbirds	*Stephanoxis lalandi, Clytolaema rubricauda, Leucochloris albicollis*	Martinelli (1994)	Visited by hummingbirds, *S. lalandi* likely the most effective vector in the study area due to its frequency and the fact of always touching the flowers stigma during the visits (Martinelli 1994).
V. atra	AF	Bats	*Anoura caudifer*	Fischer (1994), Martinelli (1994)	*A. caudifer* visited the flowers and presented pollen grains on the snout and forehead (Martinelli 1994). Glossophaginae bat visited flowers touching their reproductive parts, the pollen was deposited on its head (Fischer 1994).
V. bituminosa	AF	Bats, hummingbirds*, bees*, Diptera*	*Anoura caudifer, Ramphodon naevius, Trigona spinipes*	Sazima *et al.* (1995), Wendt *et al.* (2008), R.L.M. (personal observation)	The bat *A. caudifer* touches the reproductive organs of the flowers to feed on the nectar (Sazima 1995).

V. cacuminis	AF	Hummingbirds, bees*		R. Sadala, Museu Nacional, Universidade Federal do Rio de Janeiro, unpublished data.	Visited by hummingbirds and *Apis melifera* was registered collecting pollen without pollinate flowers.
V. carinata	AF	Hummingbirds, bees*	*Phaethornis eurynome, Ramphodon naevius*	Araujo *et al.* (1994), Machado and Semir (2006), Vizentin-Bugoni *et al.* (2014)	*R. naevius* visited and pollinated flowers touching the stigma and stamens of flowers. Halictidae bees visited the flowers but they rarely touch the stigmas, they touch the petals and usually go diretly to the anthers to collect pollen (Araujo *et al.* 1994). *P. eurynome* pollinated flowers touching their reproductive parts (Machado and Semir 2006). *P. eurynome* touched flower reproductive organs (Vizentin-Bugoni *et al.* 2014).
V. delicatula	AF	Hummingbirds*	*Ramphodon naevius, Thalurania glaucopis, Phaethornis eurynome*	Wendt et al. (2008)	Hummingbirds were recorded visiting the flowers.
V. ensiformis	AF	Hummingbirds, bees*	*Ramphodon naevius, Phaethornis eurynome, Phaethornis squalidus, Leucochloris albicollis, Melanotrochilus fuscus* (= *Florisuga fusca*), *Amazilia fimbriata*	Araujo *et al.* (1994), Martinelli (1994), Buzato *et al.* (2000), Wendt *et al.* (2008)	*L. albicolis* certainly touched the stigma during visits (Martinelli 1994). R. naevius visited and pollinated flowers touching the stigma and stamens of flowers. Halictidae bees visited the flowers but they rarely touch the stigmas, they touch the petals and usually go diret to the anthers to collect pollen (Araujo *et al.* 1994). *R. naevius* and *P. eurynome* touched the sexual parts of flowers (Buzato *et al.* 2000). *R. naevius* and *P. eurynome* were recorded visiting the flowers (Wendt *et al.* 2008).
V. erythrodactylon	AF	Hummingbirds	*Phaethornis eurynome*	Vizentin-Bugoni *et al.* (2014)	*P. eurynome* touched flower reproductive organs.
V. fenestralis	AF	Hummingbirds*	*Ramphodon naevius*	Wendt *et al.* (2008)	The hummingbird was recorded visiting the flowers.

V. flammea	AF	Hummingbirds	*Phaethornis eurynome, Leucochloris albicollis, Melanotrochilus fuscus (= Florisuga fusca), Thalurania glaucopis, Clytolaema rubricauda*	Fischer (1994), Martinelli (1994), Buzato et *al.* (2000), Machado and Semir (2006)	*M. fuscus* (= *F. fusca*) and *T. glaucopis* visited flowers, touching their reproductive parts, the pollen was deposited on their heads (Fischer 1994). *R. naevius* and *M. fuscus* (= *F. fusca*) touched the sexual parts of flowers (Buzato *et al.* 2000). *L. albicollis* and *P. eurynome* pollinated flowers touching their reproductive parts (Machado and Semir 2006).
V. friburgensis	AF	Hummingbirds, bees, other insects*	*Amazilia fimbriata, Thalurania glaucopis, Eupetomena macroura, Anthracothorax nigricollis, Xylocopa brasilianorum, Colibri coruscans*	Bernardello *et al.* (1991), Souza (2004), Schmid *et al.* (2011)	*C. coruscans* was seen feeding on flowers (Bernardello *et al.* 1991). *A. fimbriata* and *A. nigricollis* are main pollinators at the locality and *X. brasilianorum* is considered a secondary pollinator (Souza 2004). Hummingbirds and bees were more frequent visitors. Hummingbirds touched the anthers and stigma, the most frequent was *A. fimbriata*. Occasionally the bees touched the stigmas while collecting pollen. Many insects visited the flowers (Schmid *et al.* 2011).
V. gigantea	AF	Bats	*Anoura caudifer*	Sazima *et al.* (1995)	The bat touches the reproductive organs of the flowers to feed on the nectar.
V. gracilior	AF	Hummingbirds, butterflies, bees	*Ramphodon naevius, Thalurania glaucopis, Phaethornis eurynome, Phaethornis squalidus, Heliconius nattereri, Trigona fulviventris*	Varassin and Sazima (2012), Wendt *et al.* (2008)	*P. eurynome, P. squalidus, R. naevius, H. nattereri* and *T. fulviventris* pollinators carried pollen and contacted stigmas (Varassin and Sazima 2012). *R. naevius, T. glaucopis* and *P. eurynome* were recorded visiting the flowers (Wendt *et al.* 2008).
V. guttata	AF	Hummingbirds	*Phaethornis eurynome*	Kaehler *et al.* (2005)	*P. eurynome* pollinated flowers touching their reproductive parts.
V. heterostachys	AF	Hummingbirds	*Phaethornis eurynome, Leucochloris albicollis*	Martinelli (1994), Kaehler *et al.* (2005)	*P. eurynome* pollinated flowers touching their reproductive parts (Kaehler *et al.* 2005).

V. hoehneana	AF	Bats, bees*	*Anoura caudifer*, *Pygoderma bilabiatum*, *Plebeia saiqui*	Kaehler *et al.* (2005)	*A. caudifer* and *P. bilabiatum* pollinated flowers touching their reproductive parts. *P. saiqui* did not pollinate flowers but collected pollen (Kaehler *et al.* 2005).
V. incurvata	AF	Hummingbirds, bees*	*Amazilia fimbriata*, *Phaethornis eurynome*, *Melanotrochilus fuscus*, *Ramphodon naevius*	Araujo *et al.* (1994), Martinelli (1994), Buzato *et al.* (2000), Machado and Semir (2006), Silva and Piratelli (2014), Vizentin-Bugoni *et al.* (2014)	*A. fimbriata* received loaded pollen in the forehead and touched the stigma of flowers (Martinelli 1994). *R. naevius* visited and pollinated flowers touching the stigma and stamens of flowers. Halictidae bees visited the flowers but they rarely touch the stigmas, they touch the petals and usually go diret to the anthers to collect pollen (Araujo *et al.* 1994). *R. naevius* touched the sexual parts of flowers (Buzato *et al.* 2000). *P. eurynome* and *M. fuscus* pollinated flowers touching their reproductive parts (Machado and Semir 2006). *P. eurynome* pollinated the flowers (Silva and Piratelli 2014). *P. eurynome* touched flower reproductive organs (Vizentin-Bugoni *et al.* 2014).
V. longicaulis	AF	Bats, hummingbirds*	*Anoura caudifer*, *Ramphodon naevius*	Sazima *et al.* (1995), Wendt *et al.* (2008)	The bat touches the reproductive organs of the flowers to feed on the nectar (Sazima *et al.* 1995). *R. naevius* was recorded visiting the flowers (Wendt *et al.* 2008).
V. longiscapa	AF	Bats	*Anoura caudifer*	Sazima *et al.* (1995)	The bat touched the reproductive organs of the flowers to feed on the nectar.
V. minarum	CE	Hummingbirds*, wasps*		Versieux (2011)	Hummingbirds and wasps were seen visiting the flowers.
V. morreniana§	AF	Hummingbirds	*Phaethornis eurynome*	Buzato *et al.* (2000)	*P. eurynome* touched the sexual parts of flowers.

V. neoglutinosa	AF	Hummingbirds, bees, ants	*Amazilia fimbriata, Chlorostilbon aureoventris (= Chlorostilbon lucidus), Florisuga fusca, Thalurania glaucopis, Eupetomena macroura, Apis mellifera, Trigona spinipes, Lestrimelitta sp., Camponotus rufipes*	Martinelli (1994), Rodrigues (2008), Magalhães *et al.* (2018)	*A. fimbriata* seems the most effective pollinator at the study site due to the frequency and behaviour when visiting the flowers, it visited all open flowers in each individual and touched the stigma at each visit (Martinelli 1994). *A. fimbriata*, *F. fusca* and *T glaucopis* visited the flowers, *T. glaucopis* was the most frequent pollinator (Rodrigues 2008). The hummingbird *A. fimbriata*, the ant *C. rufipes* and the bee *A. melifera* are most frequent pollinators at the locality (Magalhães *et al.* 2018).
V. paraibica	AF	Hummingbirds		Martinelli (1994)	An unindentified hummingbird contacted the stigma of flowers during the visits. No other visitors were observed at the locality (Martinelli 1994).
V. philippocoburgii	AF	Hummingbirds	*Thalurania glaucopis, Phaethornis eurynome, Ramphodon naevius*	Araujo *et al.* (1994), Buzato *et al.* (2000), Machado and Semir (2006)	*P. eurynome* was recorded visiting the flowers (Araujo et al 1994). *R. naevius* touched the sexual parts of flowers (Buzato *et al.* 2000). *T. glaucopis* pollinated flowers touching their reproductive parts (Machado and Semir 2006).
V. platynema var. *platynema*	AF	Bees*		R. L. M. (personal observation)	The bees were registered visiting the flowers.
V. platynema* var. *variegata	AF	Bats, hummingbirds*, orthoptera*	*Anoura caudifer, Anoura geoffroyi, Pygoderma bilabiatum, Phaethornis eurynome, Thalurania glaucopis, Leucochloris albicollis*	Kaehler *et al.* (2005)	The bats pollinated flowers touching their reproductive parts. The hummingbirds collected nectar during the morning when flowers were withering and the orthoptera collected pollen, they were not classified as pollinators by the authors (Kaehler *et al.* 2005).

V. poenulata	AF	Hummingbirds*	*Ramphodon naevius*, *Phaethornis eurynome*	Wendt *et al.* (2008)	The hummingbirds were recorded visiting the flowers.
V. procera	AF	Hummingbirds	*Amazilia fimbriata*	Buzato *et al.* (2000)	*A. fimbriata* touched the sexual parts of flowers.
V. psittacina	AF	Hummingbirds	*Melanotrochilus fuscus*, *Phaethornis pretrei*, *Leucochloris albicollis*, *Phaethornis eurynome*, *Phaethornis squalidus*, *Ramphodon naevius*	Martinelli (1994), Siqueira-Filho and Machado (2004), Varassin and Sazima (2012).	Both hummingbirds visited flowers touching the stigma and anthers while collecting nectar, in frequent visits (Martinelli 1994). Observations of these hummingbirds were rare at the locality, additionally, *Glaucis hirsuta* and *Phaethornis ruber* were registered at the locality (Siqueira-Filho and Machado 2004). *L. albicollis*, *P. eurynome*, *P. squalidus* and *R. naevius* pollinators carried pollen and contacted stigmas (Varassin and Sazima 2012).
V. reitzii	AF	Hummingbirds	*Leucochloris albicollis*	Favretto *et al.* (2010)	Flower visited by *L. albicollis*.
V. rodigasiana	AF	Hummingbirds	*Thalurania glaucopis*, *Ramphodon naevius*, *Phaethornis eurynome*	Buzato *et al.* (2000), Rocca and Sazima (2013)	*T. glaucopis* touched the sexual parts of flowers (Buzato et al. 2000). *T. glaucopis* and *R. naevius* pollinated flowers (Rocca and Sazima 2013).
V. ruschii	AF	Bats*		Wendt *et al.* (2008)	The bat was recorded visiting the flowers .
V. sazimae	AF	Bats, hummingbirds*	*Anoura caudifer*, *Anoura geoffroyi*, *Stephanoxis lalandi*	Sazima *et al.* (1995)	The bats touch the reproductive organs of the flowers to feed on the nectar. The hummingbird visited the withered flowers during the morning to feed on the remaining nectar.
V. scalaris	AF	Hummingbirds	*Ramphodon naevius*, *Thalurania glaucopis*, *Phaethornis eurynome*	Buzato *et al.* (2000), Wendt *et al.* (2008)	*R. naevius* touched the sexual parts of flowers (Buzato *et al.* 2000). *R. naevius*, *T. glaucopis* and *P. eurynome* were recorded visiting the flowers (Wendt *et al.* 2008).

V. sceptrum	AF	Hummingbirds	*Phaethornis eurynome, Leucochloris albicollis*	Buzato *et al.* (2000)	*P. eurynome* and *L. albicolis* touched the sexual parts of flowers.
V. simplex	AF	Hummingbirds	*Ramphodon naevius, Thalurania glaucopis, Phaethornis eurynome, Trigona spinipes*	Varassin and Sazima (2012), Wendt *et al.* (2008), Vizentin-Bugoni *et al.* (2014)	*P. eurynome* pollinator carried pollen and contacted stigmas (Varassin and Sazima 2012). *R. naevius*, *T. glaucopis* and *P. eurynome* were recorded visiting the flowers (Wendt et al. 2008). *P. eurynome* touched flower reproductive organs (Vizentin-Bugoni *et al.* 2014).
V. sparsiflora	AF	Hummingbirds	*Clytolaema rubricauda, Leucochloris albicollis, Stephanoxis lalandi, Phaethornis eurynome*	Martinelli (1994)	The most effective hummingbird pollinator at the locality is *P. eurynome* based on frequency of visits and behavious. All hummingbird species where registered touching the stigma of flowers and no other visitor was recorded (Martinelli 1994).
V. haematina (=*V. triligulata*)	AF	Hummingbirds	*Stephanoxis lalandi, Clytolaema rubricauda, Leucochloris albicollis*	Martinelli (1994)	*S. lalandi* and *L. albicollis* touched the stigma and anthers of flowers during the visits and were the most frequent visitors (Martinelli 1994).
V. vagans	AF	Hummingbirds, bees*	*Ramphodon naevius, Thalurania glaucopis, Phaethornis eurynome, Phaethornis squalidus, Trigona spinipes*	Fischer (1994), Martinelli (1994), Wendt *et al.* (2008)	*T. glaucopis* visited flowers, touching their reproductive parts, the pollen was deposited on its throat (Fischer 1994). *R. naevius, T. glaucopis, P. eurynome, P. squalidus* and *T. spinipes* were recorded visiting the flowers (Wendt *et al.* 2008).

Literature Cited

Araujo AC, Fischer EA, Sazima M. 1994. Floração sequencial e polinização de três espécies de *Vriesea* (Bromeliaceae) na região de Juréia, sudeste do Brasil. *Revista Brasileira de Botânica* 17: 113–118.

Bernardello M, Galetto L, Juliani HR. 1991. Floral nectar, nectary structure and pollinators in some Argentinean Bromeliaceae. *Annals of Botany* 67: 401–411.

Buzato S, Sazima M, Sazima I. 2000. Hummingbird-pollinated floras at three Atlantic Forest sites. *Biotropica* 32: 824–841.

Costa AF. 1997. Nota sobre o Herbário e o Jardim Botânico da Universidade de Liège, Bélgica: a importância das coleções e o exemplo de *Vriesea morreniana*. *Bromélia* 4: 9–13.

Favretto MA, Hoeltgebaum MP, Lingnaur R, D'Agostini FM. 2010. Beija-flores visitantes de bromélias no Parque Natural Municipal Rio do Peixe, Joaçaba, Santa Catarina, Brasil. *Atualidades Ornitológicas* 158: 11–13.

Fischer EE. 1994. *Polinização, fenologia e distribuiçõ espacial de Bromeliaceae numa comunidade de Mata Atlântica, litoral Sul de São Paulo.* Master dissertation, Universidade Estadual de Campinas, Brazil.

Kaehler M, Varassin IG, Goldenberg R. 2005. Polinização em uma comunidade de bromélias em Floresta Atlântica Alto-montana no Estado do Paraná, Brasil. *Revista Brasileira de Botânica* 28: 219–228.

Machado CG, Semir J. 2006. Fenologia da floração e biologia floral de bromeliáceas ornitófilas de uma área da Mata Atlântica do Sudeste brasileiro. *Revista Brasileira de Botânica* 29: 163–174.

Magalhães AFP, Maruyama PK, Tavares LAF, Martins RL. 2018. The relative importance of hummingbirds as pollinators in two bromeliads with contrasting floral specializations and breeding systems. *Botanical Journal of the Linnean Society* 188: 316–326.

Martinelli G. 1994. *Reproductive biology of Bromeliaceae in the Atlantic rainforest of southeastern Brazil*. PhD thesis, University of St Andrews, Scotland, UK.

Nunes CE, Briet J, Galetto L, Sazima M, Amorim FW. 2018. Nectar ecology of the endemic epiphytic hummingbird-pollinated bromeliad *Vriesea altodaserrae*: secretion dynamics and pollinator visitation pattern. *Acta Botanica Brasilica* 32: 479–486.

Rocca MA, Sazima M. 2013. Quantity versus quality: identifying the most effective pollinators of the hummingbird-pollinated *Vriesea rodigasiana* (Bromeliaceae). *Plant Systematics and Evolution* 299: 97–105.

Rodrigues MS. 2008. *Partilha de recursos florais por beija-flores em uma área de Mata Atlântica na Ilha de Marambaia, RJ*. Master dissertation, Universidade Federal Rural do Rio de Janeiro, Brazil.

Sazima M, Buzato S, Sazima I. 1995. Bat pollination of *Vriesea* in southeastern Brazil. *Bromélia* 2: 29–37.

Schmid S, Schmid VS, Zillikens A, Steiner J. 2011. Diversity of flower visitors and their role for pollination in the ornithophilous bromeliad *Vriesea friburgensis* in two different habitats in southern Brazil. *Ecotropica* 17: 91–102.

Silva BG, Piratelli AJ. 2014. Foraging behaviour of the scale-throated hermit *Phaethornis eurynome* Lesson, 1832 (Aves, Trochilidae) in *Vriesea incurvata* Gaudich (Bromeliaceae). *Brazilian Journal of Biology* 74: 311–314.

Siqueira-Filho JA, Machado IC. 2004. Síndromes de polinização de uma comunidade de Bromeliaceae e biologia floral de *Vriesea psittacina* (Hooker) Lindley (Bromeliaceae) em Brejo dos Cavalos, Caruaru, Pernambuco. In: Porto KC, Cabral JJP, Tabarelli M orgs. *Brejos de Altitude em Pernambuco e Paraíba- História Natural, Ecologia e Conservação*. Brasília, Ministério do Meio Ambiente, 227–284.

Souza DAS. 2004. *Ecologia reprodutiva de Vriesea friburgensis Mez var. paludosa (L. B. Smith) L. B. Smith (Bromeliaceae), em área de restinga, no litoral sul de Santa Catarina, Brasil*. Master dissertation, Universidade Federal de Santa Catarina, Brazil.

Varassin IG, Sazima M. 2012. Spatial heterogeneity and the distribution of bromeliad pollinators in the Atlantic Forest. *Acta Oecologica* 43, 104–112.

Versieux LM. 2011. Brazilian plants urgently needing conservation: the case of *Vriesea minarum* (Bromeliaceae). *Phytotaxa* 28: 35–49.

Vizentin-Bugoni J, Maruyama PK, Sazima M. 2014. Processes entangling interactions in communities: forbidden links are more important than abundance in a hummingbird–plant network. *Proceedings of the Royal Society B: Biological Sciences* 281: e20132397.

Wendt T, Coser TS, Matallana G, Guilherme FAG. 2008. An apparent lack of prezygotic reproductive isolation among 42 sympatric species of Bromeliaceae in southeastern Brazil. *Plant Systematics and Evolution* 275: 31.

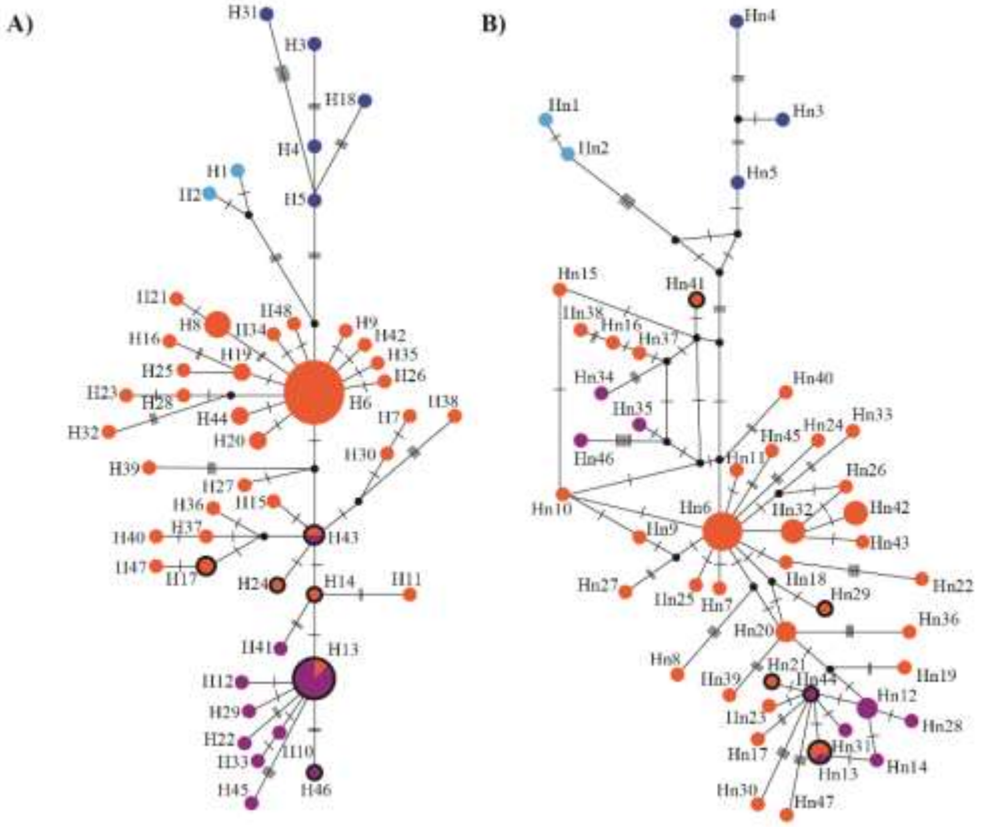

Figure S1. (A) cpDNA (*matK* and *rps16-trnK*) and (B) *PHYC* median joining networks showing genetic divergence among *Vriesea* pollination groups. Colors represent hummingbird (orange) and bat (purple) syndromes. The outgroup *Alcantarea* and *Stigmatodon* is shown in blue. Each circle represents a haplotype with the size proportional to its total frequency. Haplotypes of *Vriesea* species with floral traits distinct from the typical hummingbird and bat-pollinated flowers (mixed floral types) are circled in black. Mutational steps are indicated with dashes and hypothetic haplotypes with black dots. See codes in Table 1.

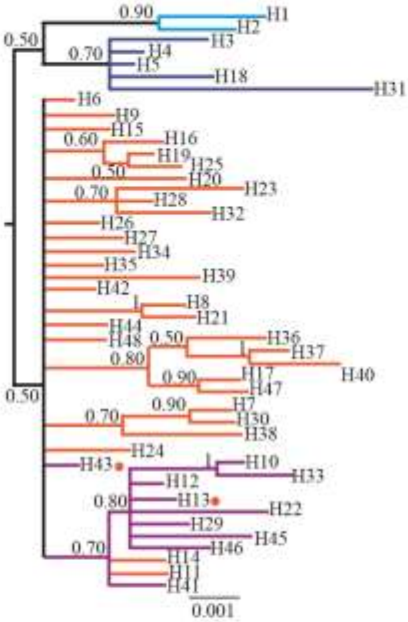

Figure S2. Bayesian phylogeny of cpDNA haplotypes (*matK* and *rps16-trnK*) for 83 taxa, including 76 *Vriesea* accessions. Posterior probabilities above 0.50 are shown. Colors represent hummingbird (orange) and bat (purple) syndromes in *Vriesea*. The outgroup *Alcantarea* and *Stigmatodon* is indicated in blue. The phylogeny of cpDNA haplotypes was generally unresolved. Haplotypes from genus *Vriesea* formed a clade poorly supported (PP = 0.50). Five hummingbird-pollinated clades emerged, including the highly supported *V. corcovadensis* group (H17- 47, PP = 0.90). Another clade of bat-pollinated species was resolved (H10-11-12-13-14-22-29-33-41-45-46, PP=0.70), but also included *V. botafogensis*, *V. billbergioides* and *V. cacuminis* (hummingbird-pollinated). See codes in Table 1.

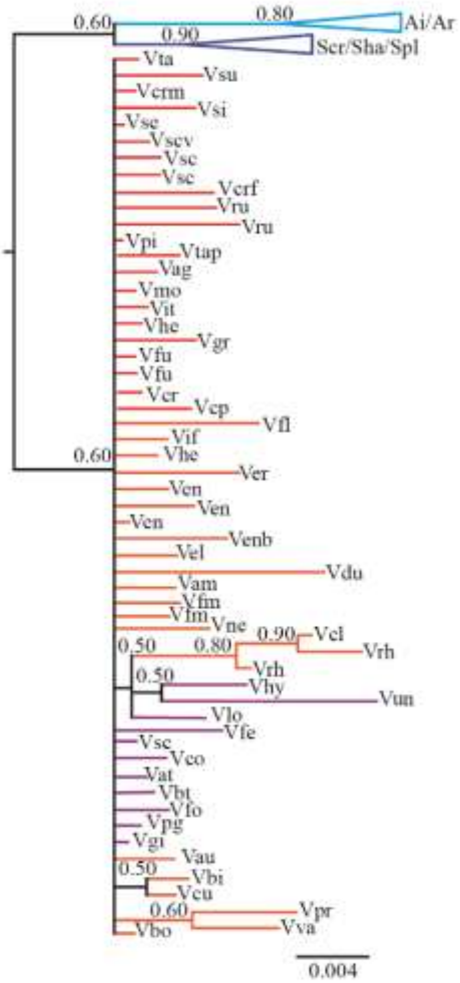

Figure S3: Bayesian phylogeny based on *PHYC* dataset for 60 taxa, including 55 *Vriesea* accessions. Posterior probabilities above 0.50 are shown. Colors represent hummingbird (orange) and bat (purple) syndromes in *Vriesea*. The outgroup *Alcantarea* and *Stigmatodon* is indicated in blue. *Vriesea* is poorly supported as monophyletic (PP = 0.60). The few clades with support higher than 0.50 PP constitute groupings of morphologically similar species within each syndrome. A clade comprising *V. rhodostachys* (Vrh) and *V. calimaniana* (Vcl) form a geographic group as they occur in adjacent areas (PP = 0.80). See codes in Table 1.

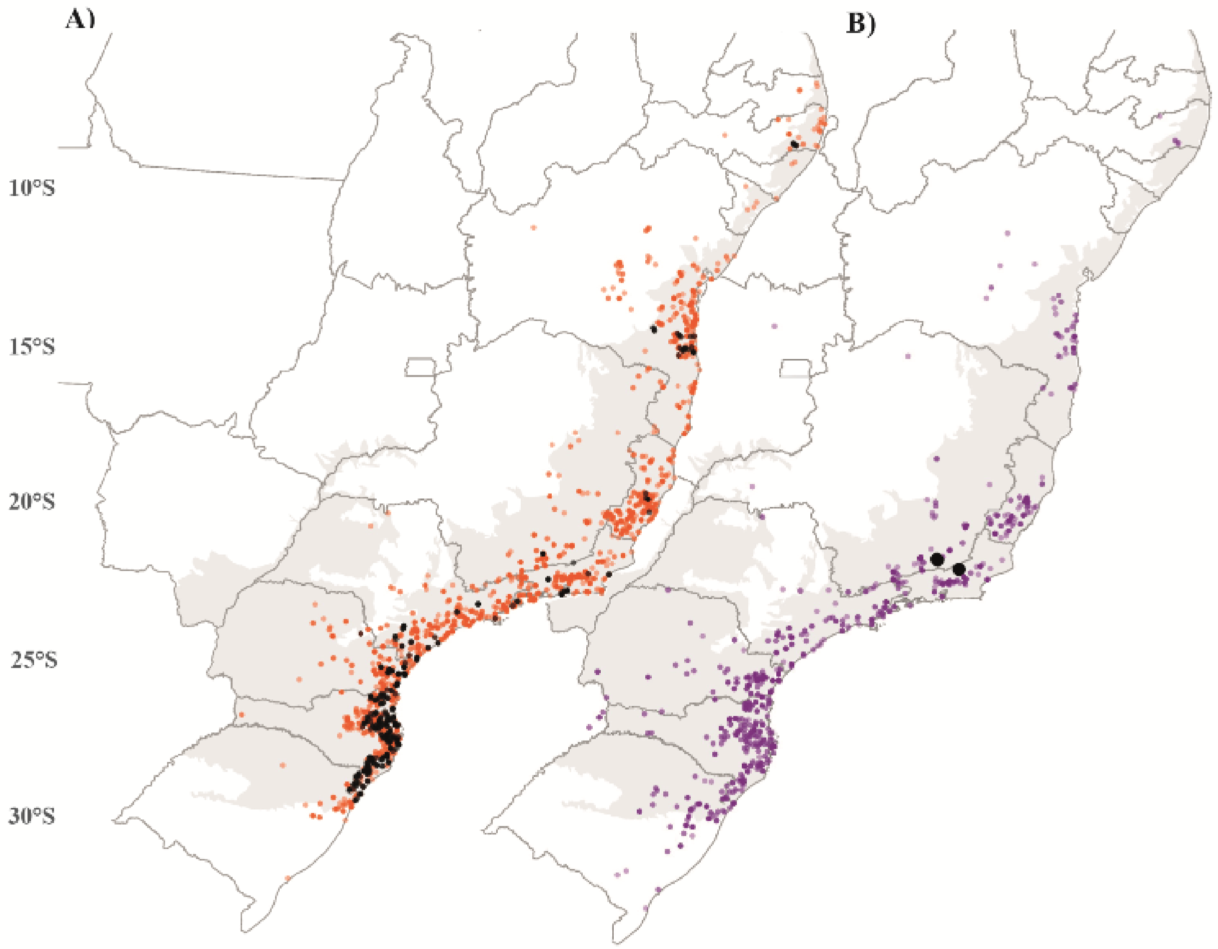

Figure S4: Map of Brazil with geographic distribution of (A) hummingbird (orange) and (B) bat-pollinated (purple) *Vriesea* species along the Atlantic Forest (in gray). Species with floral traits distinct from the typical hummingbird and bat-pollinated flowers (mixed floral types) in black circles. Records from the dataset compiled in Ramos *et al*. (2018) for the Atlantic Forest epiphytes, complemented with our personal collections and the taxonomic revisions of Moura (2011) and Uribbe *et al*. (2020).

Literature Cited

Moura RL. 2011. Revisão Taxonômica do Grupo *Vriesea platynema* Gaudich. (Bromeliaceae). Tese de Doutorado. Universidade Federal do Rio de Janeiro, Rio de Janeiro. 194p.

Ramos FN, Mortara SR, Monalisa-Francisco N, *et al.* 2019. Atlantic epiphytes: a data set of vascular and non-vascular epiphyte plants and lichens from the Atlantic Forest. *Ecology* 100: e02541.

Uribbe FP, Neves B, Jacques, SSA, Costa AF. 2020. Morphological variation in the *Vriesea procera* complex (Bromeliaceae, Tillandsioideae) in the Brazilian Atlantic Rainforest, with recognition of new taxa. *Systematic Botany* 45: 53–68.
